# Dynamics of the leaf endoplasmic reticulum modulate β-glucosidase-mediated stress-activated ABA production from its glucosyl ester

**DOI:** 10.1093/jxb/erz528

**Published:** 2019-11-25

**Authors:** Yiping Han, Shunsuke Watanabe, Hiroshi Shimada, Atsushi Sakamoto

**Affiliations:** 1 Graduate School of Science, Hiroshima University, Higashi-Hiroshima, Japan; 2 RIKEN Center for Sustainable Resource Science, Tsurumi-ku, Yokohama, Japan; 3 Graduate School of Integrated Sciences for Life, Hiroshima University, Higashi-Hiroshima, Japan; 4 Hong Kong Baptist University, Hong Kong

**Keywords:** Abscisic acid (ABA), ABA conjugate, allantoin, *Arabidopsis thaliana*, β-glucosidase, drought, dehydration, endoplasmic reticulum (ER), ER body, ER dynamics, stress response

## Abstract

The phytohormone abscisic acid (ABA) is produced via a multistep *de novo* biosynthesis pathway or via single-step hydrolysis of inactive ABA-glucose ester (ABA-GE). The hydrolysis reaction is catalyzed by β-glucosidase (BG, or BGLU) isoforms localized to various organelles, where they become activated upon stress, but the mechanisms underlying this organelle-specific activation remain unclear. We investigated the relationship between the subcellular distribution and stress-induced activation of BGLU18 (BG1), an endoplasmic reticulum enzyme critical for abiotic stress responses, in *Arabidopsis thaliana* leaves. High BGLU18 levels were present in leaf petioles, primarily in endoplasmic reticulum bodies. These Brassicaceae-specific endoplasmic reticulum-derived organelles responded dynamically to abiotic stress, particularly drought-induced dehydration, by changing in number and size. Under stress, BGLU18 distribution shifted toward microsomes, which was accompanied by increasing BGLU18-mediated ABA-GE hydrolytic activity and ABA levels in leaf petioles. Under non-stress conditions, impaired endoplasmic reticulum body formation caused a microsomal shift of BGLU18 and increased its enzyme activity; however, ABA levels increased only under stress, probably because ABA-GE is supplied to the endoplasmic reticulum only under these conditions. Loss of BGLU18 delayed dehydration-induced ABA accumulation, suggesting that ABA-GE hydrolysis precedes the biosynthesis. We propose that dynamics of the endoplasmic reticulum modulate ABA homeostasis and abiotic stress responses by activating BGLU18-mediated ABA-GE hydrolysis.

## Teaser

In Arabidopsis leaves, the stress-induced production of abscisic acid from its inactive glucose conjugate involves dynamic changes in the endoplasmic reticulum.

## Introduction

The plant hormone abscisic acid (ABA) is a key regulator of various physiological and developmental processes (Zeevaart and Creelman, 1988; Zhu, 2002). When plants are exposed to unfavorable environmental conditions such as drought, cellular ABA levels increase rapidly, inducing a wide array of physiological responses to protect plants from water loss and damage (Schroeder *et al.*, 2001; Shinozaki and Yamaguchi-Shinozaki, 2007). Stress-induced ABA accumulation is regulated by the precise balance between its biosynthesis, catabolism, and reversible conjugation (Nambara and Marion-Poll, 2005). The *de novo* biosynthesis of ABA requires multistep enzymatic reactions that initially occur in plastids. These reactions convert the C_40_ carotenoid zeaxanthin to xanthoxin, the direct C_15_ precursor of ABA (Marin *et al.*, 1996; Schwartz *et al.*, 1997; Xiong *et al.*, 2002), which is oxidized to ABA after export to the cytosol (Seo *et al.*, 2000; Cheng *et al.*, 2002; González-Guzmán *et al.*, 2002). Inactivation of ABA occurs in the endoplasmic reticulum (ER), where the hormone is irreversibly catabolized to phaseic acid and then to dihydrophaseic acid or its epimer (Kushiro *et al.*, 2004; Saito *et al.*, 2004; Saika *et al.*, 2007). ABA is also inactivated by direct conjugation with UDP-glucose to form ABA-glucosyl ester (ABA-GE) (Lim *et al.*, 2005; Priest *et al.*, 2006), which is generally found in vacuoles or the apoplast (Harris and Dugger, 1986; Dietz *et al.*, 2000).

Although ABA-GE was long regarded as a simple byproduct of ABA catabolism, recent reverse genetic studies have revealed that it constitutes an inactive pool of ABA that serves as an alternative source of ABA production during physiological responses to environmental stimuli and stresses (Lee *et al.*, 2006; Xu *et al.*, 2012; Li *et al.*, 2013; Watanabe *et al.*, 2014*b*; Ondzighi-Assoume *et al.*, 2016). In Arabidopsis (*Arabidopsis thaliana*), ABA-GE is hydrolyzed to release free ABA via a single-step reaction catalyzed by two β-glucosidase (BG, or BGLU) isoforms known as BG1 or BGLU18 (Lee *et al.*, 2006) and BG2 or BGLU33 (Xu *et al.*, 2012). BGLU18 is localized to the ER, whereas BGLU33 is a vacuolar enzyme. The loss-of-function *bg1* and *bg2* mutants exhibit enhanced sensitivity to drought and salt stress, and the *bg1 bg2* double mutants show additive effects. In terms of ABA homeostasis and responses, *bg1* exhibits more severe phenotypes, including decreased ABA levels, early germination, and impaired stomatal modulation. Conversely, overexpressing either enzyme alone confers significant salt tolerance in Arabidopsis. It is thought that ABA-GE hydrolysis contributes to rapid and local ABA release upon the onset of stress.

The activities of both BGLU18 and BGLU33 increase in response to dehydration. However, the mechanistic aspects behind the activation of these enzymes appear to differ. BGLU18 normally occurs as monomers that undergo multimerization, and thereby activation, in response to drought stress (Lee *et al.*, 2006; Watanabe *et al.*, 2014*b*), whereas BGLU33 normally exists as active, stable multimers whose levels increase upon exposure to stress (Xu *et al.*, 2012). The different subcellular localizations of the two enzymes might, at least in part, underlie the difference in their activation mechanisms. For example, under normal conditions, BGLU33 is maintained at low levels in vacuoles, where it is presumably degraded continuously by proteases (Xu *et al.*, 2012). We previously demonstrated that independently of stress, BGLU18 is activated by the accumulation of allantoin, a major intermediate of purine catabolism, leading to increased ABA levels and enhanced tolerance to drought and osmotic stress (Watanabe *et al.*, 2014*b*). In plants, allantoin is generated from oxopurine uric acid in peroxisomes and transported to the ER, where it is metabolized under normal conditions or accumulates under stress (Werner and Witte, 2011). These findings raise the possibility that ER-associated cellular processes are involved in BGLU18 activation, thereby enhancing its catalytic reactions, including ABA production.

The ER forms highly organized network structures that are extremely dynamic, contributing greatly to its functional versatility, which includes roles in cellular signaling, metabolism, and the storage, transport, and secretion of proteins (Vitale and Denecke, 1999; Schwarz and Blower, 2016). Interestingly, in plants of the Brassicales order, including Arabidopsis, the ER develops unique spindle-shaped compartments called ER bodies, which are studded with ribosomes and are typically ~5–10 μm long and 0.5–1 μm wide (Hayashi *et al.*, 2001; Nakano *et al.*, 2014). This subdomain of the ER was originally identified in epidermal cells of Arabidopsis cotyledons and is ubiquitous in young seedlings and the roots of mature plants. In Arabidopsis, the major component of ER bodies is BGLU. ER bodies in Arabidopsis roots contain large amounts of BGLU23 (also known as PYK10), which possesses myrosinase activity (Matsushima *et al.*, 2003; Nakano *et al.*, 2017). BGLU23 and its homologs are thought to function in plant defense against biotic stresses such as pest and pathogen attack (Sherameti *et al.*, 2008; Yamada *et al.*, 2011). Few ER bodies are present in the aerial parts of Arabidopsis plants, but their formation is induced upon mechanical wounding or treatment with the plant defense signaling molecule methyl jasmonate (Matsushima *et al.*, 2002). In contrast to constitutively occurring ER bodies, which harbor BGLU23, these inducible ER bodies exclusively accumulate BGLU18 (Ogasawara *et al.*, 2009), suggesting that ER bodies in shoots might function in BGLU18-mediated stress responses, including stress-induced ABA production. However, to date, this possibility has not been examined, and the roles of ER bodies in ABA homeostasis and metabolism remain unknown.

Here, to address these questions, we analyzed the distribution and ABA-GE hydrolysis activity of BGLU18 in Arabidopsis leaves exposed to abiotic stress, particularly drought-induced dehydration, in relation to the behavior of ER and ER bodies. Our findings demonstrate that dynamic changes in ER status are involved in the rapid activation of BGLU18 in response to dehydration stress, providing unique mechanistic insights into the production of ABA from its inactive glucose conjugates under stress.

## Materials and methods

### Plant materials and growth conditions

Arabidopsis (*A. thaliana* [L.] Heynh.) accession Columbia-0 was used as the wild type (WT). The following mutant and SALK T-DNA insertion lines were obtained from the Arabidopsis Biological Resource Center (Ohio State University, Columbus, OH, USA): *aba deficient2-1* (*aba2-1*; CS156; Léon-Kloosterziel *et al.*, 1996) for *ABA2* (At1g52340), *allantoinase-1* (*aln-1*; SALK_000325; Watanabe *et al.*, 2014b) for *ALLANTOINASE* (At4g04955), *bglu18* (SALK_075731C; Ogasawara *et al.* 2009) for *BGLU18* (At1g52400), and *nai2-2* (SALK_005896; Yamada *et al.*, 2008) for *NAI2* (At3g19590). The *bglu18 nai2-2* double mutant was obtained by crossing the respective single mutants, and the *aln-1 bglu18* double mutant was described previously (Takagi *et al.*, 2016). Two transgenic lines, *GFP-h* and *GFP-h nai2-2*, which express green fluorescent protein (GFP) with an ER retention signal, HDEL (GFP-h), in the WT and *nai2-2* backgrounds, respectively, were described by Hayashi *et al.* (2001) and Yamada *et al.* (2008). These *GFP-h* lines were crossed with some of the above-mentioned mutants to allow the ER/ER bodies to be visualized in each genetic background. PCR genotyping was performed to confirm the genotypes of the established lines using T-DNA and gene-specific primers (Supplementary Table S1 at *JXB* online).

Surface-sterilized seeds were sown on Petri plates containing half-strength Murashige and Skoog basal salt medium with vitamins supplemented with 1% (w/v) sucrose and solidified with 0.3% (w/v) Gellan Gum (Wako Pure Chemical Industries, Ltd, Osaka, Japan). After incubation at 4 °C for 2 d, the plates were placed in a growth cabinet at 22 °C under 60–70 μmol photons m^–2^ s^–1^ of light with a 16 h photoperiod provided by white fluorescent lamps, and 14- or 16-day-old plants were used for all experiments.

### Protein extraction and immunoblotting

Rosette leaves from 14-day-old plants were divided into leaf blades and petioles. Each leaf part was homogenized in 50 mM sodium phosphate buffer (pH 7.0) containing 150 mM NaCl, 0.02% (w/v) NaN_3_, 10 mM DTT, and 0.1% (v/v) Triton X-100. An aliquot of the resulting protein extract was separated by SDS–PAGE using a 10% SDS gel and transferred onto a polyvinylidene difluoride membrane (Immobilon-P; Millipore, Billerica, MA, USA). After blocking with 3% (w/v) fat-free skimmed milk, the blotted membrane was incubated with the primary antibodies anti-BGLU18 (Ogasawara *et al.*, 2009), anti-NAI2 (Yamada *et al.*, 2008), anti-binding protein (BiP; Yamada *et al.*, 2008) (each at 1:5000 dilution), and anti-GFP antibody (Abcam, Tokyo, Japan) (at 1:10 000 dilution). The membrane was incubated with anti-rabbit IgG secondary antibodies conjugated to horseradish peroxidase at 1:20 000 dilution. Chemiluminescent immunoblotting was performed using a Western Lighting Plus-ECL kit (Perkin-Elmer Life Sciences, Wellesley, MA, USA), and the signals were digitally captured on a VersaDoc 5000 imaging system using Quantity One software (Bio-Rad Laboratories, Hercules, CA, USA) and analyzed using ImageJ software (National Institute of Health, Bethesda, MD, USA) to determine the relative intensities of the protein bands.

### Subcellular fractionation

The fractionation scheme followed that of Matsushima *et al.* (2003) with slight modifications. Shoots of 16-day-old plants were cut on ice with a razor blade and homogenized in three volumes (v/w) of ice-cold chopping buffer containing 50 mM HEPES-NaOH (pH 7.5), 5 mM EDTA, 0.4 M sucrose, and SIGMAFAST Protease Inhibitor Tablets (one tablet per 50 ml; Sigma-Aldrich, St. Louis, MO, USA). The homogenate was passed through four layers of gauze, and the resulting filtrate (designated as the total extract) was separated into four fractions by differential centrifugation as follows. The total extract (1 ml) was centrifuged at 1000 *g* for 20 min and the pellet was saved as the P1 fraction. The supernatant was re-centrifuged at 8000 *g* for 20 min to separate the pellet (P8 fraction) from the supernatant, which was subjected to ultracentrifugation at 100 000 *g* for 60 min to obtain the microsomal pellet and soluble supernatant (P100 and S100 fractions, respectively). All centrifugation was performed at 4 °C. Each pellet was resuspended in 500 μl of chopping buffer, and the fractions were loaded on a volume to volume basis (30 μl) onto an SDS–PAGE gel for immunoblotting.

### Transient expression assays

A fusion gene between *BGLU18* and *monomeric red fluorescent protein* (*mRFP*) was constructed based on its *GFP* counterpart as described in Lee *et al.* (2006). DNA sequences encoding the signal peptide region (amino acid residues 1–39) and mature polypeptide region (residues 40–528) of BGLU18 were separately PCR-amplified from the corresponding cDNA (pda05953; provided by RIKEN BioResource Center, Tsukuba, Japan) and translationally fused to the 5′ and 3′ ends, respectively, of the coding sequence of mRFP (Supplementary Fig. S1; for primers, see Supplementary Table S1). The resulting chimeric *mRFP*-*BGLU18* gene was cloned into the pENTR vector (Thermo Fisher Scientific, San Jose, CA, USA) and, after sequence verification, transferred into pUGW2 (Nakagawa *et al.*, 2007) driven by the *Cauliflower mosaic virus 35S RNA* promoter. The resulting plasmid was introduced into leaf petioles of 14-day-old *GFP-h* plants by particle bombardment (PDU-1000/He, Bio-Rad Laboratories). The bombarded samples were incubated at 22 °C in the dark for 18 h and analyzed by confocal laser-scanning fluorescence microscopy (Fluoview FV1000D; Olympus, Tokyo, Japan). GFP and mRFP fluorescence were detected at 485–535 nm and 585–650 nm, respectively, following excitation with 473 nm or 559 nm diode lasers.

### Stress treatments and relative water content (RWC) measurements

Stress treatments were applied to plants grown aseptically in Petri plates for 14 d or 16 d. Drought-induced dehydration stress was induced by removing the lids from the plates for the indicated periods of time under aseptic conditions on a laminar flow hood (Nanjo *et al.*, 1999). For osmotic stress, plants were transferred onto solid medium containing polyethylene glycol (PEG) (mol. wt 8000; Sigma-Aldrich) to impose a low water potential of approximately –0.5 MPa, followed by incubation for 12 h (Watanabe *et al.*, 2014*b*). Salt stress was imposed by transferring the plants onto solid medium containing 150 mM NaCl and incubating them for 12 h. As a control, plants were transferred to standard medium containing no additives. After stress treatment, the plants were immediately subjected to further analysis or stored at –80 °C.

Leaf RWC was measured as described by Barrs and Weatherley (1962). Primary and secondary leaves were collected from at least 10 plants and immediately weighed to determine fresh weight (FW), followed by rehydration by floating on water for 3 h to determine turgid weight (TW). Dry weight (DW) was recorded after drying these samples at 80 °C to a constant weight. RWC was calculated using the following formula: RWC=(FW−DW)/(TW−DW)×100.

### RNA extraction and reverse transcription–quantitative PCR (RT–qPCR)

RNA extraction and RT–qPCR were performed as described (Watanabe *et al.*, 2014*b*). Briefly, total RNA was extracted from the aerial parts of plants using a NucleoSpin RNA kit (Macherey-Nagel GmbH & Co, Düren, Germany), and 1 μg of RNA was reverse-transcribed into cDNA using a ReverTra Ace qPCR RT kit (Toyobo, Osaka, Japan). qPCR was carried out with a KAPA SYBR FAST qPCR Kit (Kapa Biosystems, Inc., Woburn, MA, USA) in a 20 μl reaction containing 1× Master Mix, 0.2 μM forward and reverse primers, and 10 ng of cDNA. The thermal cycling conditions were 95 °C for 3 min and 40 cycles of 95 °C for 15 s/65 °C for 40 s, followed by 65–95 °C melting curve analysis with 0.5 °C increments. The relative transcript levels of target genes were calculated using the comparative *C*_T_ method (Livak and Schmittgen, 2001) after normalization to *SAND FAMILY PROTEIN* (*SAND*; At2g28390), *E2 UBIQUITIN-CONJUGATING ENZYME 9* (*UBC9*; At4g27960), or *PENTATRICOPEPTIDE REPEAT* (*PPR*; At1g62930) as a reference. The primer sequences for the target and reference genes are listed in Supplementary Table S1.

### Microscopic observation and analysis of ER body number and size

ER bodies, as visualized by the expression of GFP-h (Hayashi *et al.*, 2001), were detected by fluorescence microscopy using the FV1000D system as described above. To estimate the number of ER bodies, confocal microscopic images of a petiole sample were acquired at a 2 μm interval from the first (surface) to 30th (inside) layer in *z*-stack direction (60 μm in depth), and the 30 images obtained were merged using Olympus Fluoview software. A merged *z*-stack typically contained 12–15 cells with GFP-visualized ER bodies. The areas of the individual cells were measured using ImageJ, and the number of ER bodies in each cell was counted manually.

For time-course analysis of ER body number and size in dehydration-stressed plants, GFP fluorescence was observed in petiole samples under an LSM700 confocal laser-scanning microscope (Carl Zeiss, Jena, Germany) using a 488 nm diode laser line and a 490–530 nm detection band. All images were taken using the same settings: laser output strength, 22%; magnification, 40-fold; resolution, 512×512 pixels/112.5×112.5 μm; pinhole, 100 μm; gain parameters, 550; and 12-bit coloring. The region of interest was selected manually as a window of 512×512 pixels, and fluorescence analysis was performed using ImageJ following the procedure of Nagano *et al.* (2009), with the threshold set from 40 to 255 for particle analysis.

### ABA measurement

ABA was purified and quantified as described (Preston *et al.*, 2009) with slight modifications. Plant tissues were frozen in liquid nitrogen and crushed using a steel-bead homogenizer (Tissue Lyser II; Qiagen, Hilden, Germany). The resulting powder was suspended and incubated for 1 h in extraction buffer (80% acetonitrile and 1% acetic acid, v/v, in ultrapure water) that included 3′,5′,5′,7′,7′,7′-hexadeuterated ABA (d_6_-ABA; Santa Cruz Biotechnology, Santa Cruz, CA, USA) as an internal control. A clear extract was obtained by centrifugation, and the residues were re-extracted with extraction buffer without d_6_-ABA. Both extracts were combined and pre-concentrated by solid phase extraction using Oasis HLB and MCX cartridges (1 ml/30 mg, 30 μm particle size; Waters Corporation, Milford, MA, USA) before being subjected to further purification and analysis by LC-electrospray ionization-tandem MS (LC-ESI-MS/MS).

ABA was measured by LC-ESI-MS/MS according to Almeida *et al.* (2014) with minor modifications. LC separation was carried out with a Thermo ACCELA Pump coupled to an ACCELA Autosampler (Thermo Fisher Scientific) equipped with a Gemini C18 column (Gemini 5 μm C18 110 Å, 150×2 mm; Phenomenex, Torrance, CA, USA). The LC column was eluted at 25 °C with a solvent system consisting of 0.01% (v/v) acetic acid in ultrapure water (solvent A) and 0.05% (v/v) acetic acid in acetonitrile (solvent B), using a linear gradient of solvent B in solvent A from 30% to 65% over 24 min at a constant flow rate of 300 μl min^–1^. ESI-MS/MS was performed using an LTQ Orbitrap XL mass spectrometer (Thermo Fisher Scientific) with the operation parameters described in Supplementary Table S2.

### Measurement of ABA-GE hydrolysis activity

ABA-GE hydrolysis activity in microsomal fractions (P100 as described above) was assayed as described previously (Watanabe *et al.*, 2014*b*). The pelleted microsomal fractions were resuspended in ice-cold buffer containing 25 mM HEPES (pH 7.0), 250 mM sucrose, 10 mM MgCl_2_, 1 mM DTT, and 1% (v/v) Triton X-100, and aliquots of the sample were incubated in 100 nM ABA-GE (OlChemim Ltd, Olomouc, Czech Republic) at 37 °C for 1 h. The liberated ABA was recovered by solid-phase extraction and quantified by LC-ESI-MS/MS as described above.

### Statistical analysis

Results are presented as means with SD from at least three independent experiments, unless otherwise noted. Statistical significance of differences between two groups was determined using unpaired Student’s *t*-test after comparing the variances of the samples by *F*-test. Comparisons among three or more groups were performed using one-way ANOVA with Tukey’s multiple comparison test. Linear regression analysis was applied to examine the correlation between RWC and time following stress treatment.

## Results

### BGLU18 is abundant in leaf petioles and is predominantly localized to ER bodies

**Fig. 1.**
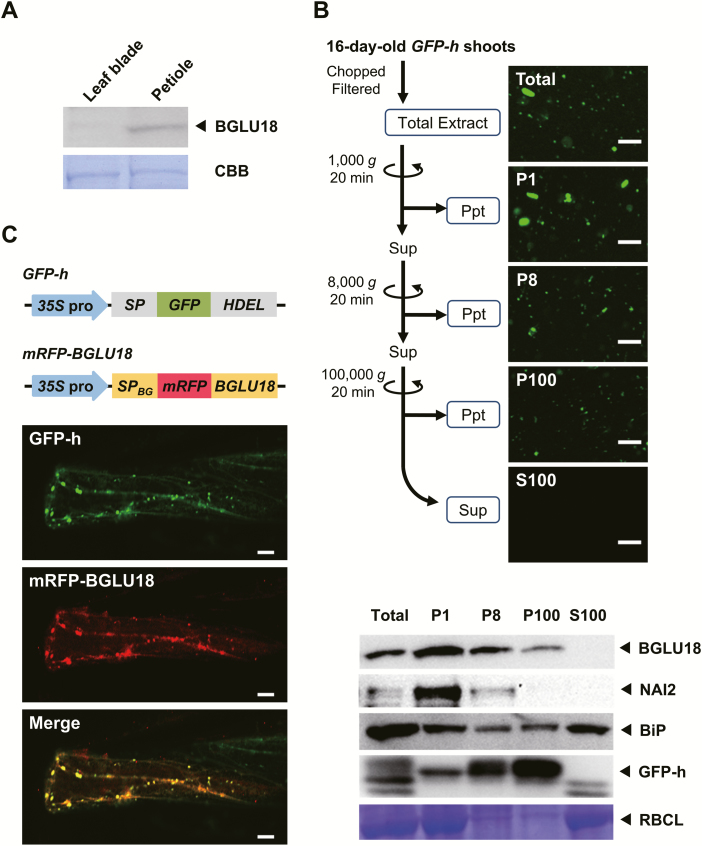
Tissue distribution and subcellular localization of BGLU18 in Arabidopsis leaves. (A) Tissue distribution within true leaves. Immunoblotting analysis was performed with anti-BGLU18 antibodies using 30 μg of total protein extracted from leaf blades and petioles of 14-day-old plants grown under normal conditions. (B) Subcellular distribution of BGLU18 as determined by biochemical fractionation. The upper panel shows the fractionation scheme and typical fluorescence images of subcellular fractions prepared from shoot extracts of *GFP-h* plants. P1, P8, and P100 indicate pellets (Ppt) obtained after centrifugation at 1000, 8000, and 100 000 *g*, respectively, and S100 indicates the supernatant (Sup) after centrifugation at 100 000 *g*. Scale bars=20 μm. The lower panel shows typical immunoblotting results for the distribution of BGLU18, NAI2, BiP, and GFP-h in the subcellular fractions. The large subunit of RBC (RBCL) was stained with Coomassie brilliant blue (CBB). Analyses were conducted on a volume to volume basis. (C) Subcellular localization of the mRFP–BGLU18 fusion protein, as determined by confocal laser-scanning fluorescence microscopy. Above, *GFP* and *mRFP* fusion constructs used to visualize ER/ER bodies and BGLU18, respectively. *35S* pro, *Cauliflower mosaic virus 35S* promoter; *SP*, signal peptide from pumpkin 2S albumin; *GFP*, green fluorescent protein; *HDEL*, ER retention signal; *SP*_*BG*_, signal peptide from BGLU18; *mRFP*, monomeric red fluorescent protein; *BGLU18*, mature polypeptide of BGLU18. Below, representative fluorescence images of leaf petiole epidermal cells of a *GFP-h* plant transiently expressing *BG1-mRFP* by particle bombardment. Scale bars=20 μm. All experiments were repeated three times to confirm the reproducibility of the results, and one representative result is shown.

*BGLU18* mRNA is expressed at high levels in the aerial parts of plants, particularly vegetative leaves (Xu *et al.*, 2004; Lee *et al.*, 2006); this finding is supported by publicly available microarray data from the Arabidopsis eFP Browser (Supplementary Fig. S2; Winter *et al.*, 2007). However, few studies have examined the protein level of BGLU18, except for an experiment in which a *BGLU18* transgene was ectopically expressed in transgenic Arabidopsis under a strong constitutive promoter (Lee *et al.*, 2006). We examined the distribution of BGLU18 in the above-ground tissues (mostly leaf blades and petioles) of 14-day-old Arabidopsis plants grown under normal conditions. Immunoblotting analysis detected more BGLU18 in petioles than in leaf blades (Fig. 1A), which is consistent with the quantitative expression data (Supplementary Fig. S3).

To assess the subcellular localization of BGLU18, we performed subcellular fractionation and immunoblotting using leaf tissues from *GFP-h* plants that stably produced GFP with an ER retention signal (Hayashi *et al.*, 2001). We mainly detected BGLU18 in the P1 and P8 fractions, which were enriched for ER bodies, as well as small amounts in the microsomal P100 fraction (where, as expected, GFP-h was most abundant), but not in the soluble S100 fraction (Fig. 1B). The BGLU18 levels in the fractionated samples were intermediate between those of the canonical ER body-specific protein NAI2 and the major ER lumen protein BiP. To obtain independent evidence for this localization pattern, we transiently expressed an *mRFP-BGLU18* translational fusion construct in the petiole tissues of *GFP-h* plants. In the transformed plants, most mRFP signals overlapped with those of GFP, representing the ER and ER bodies (Fig. 1C). Therefore, both of these approaches demonstrated that under normal conditions, BGLU18 primarily occurs as a component of ER bodies and to a minor extent in the ER in leaf petioles.

**Fig. 2.**
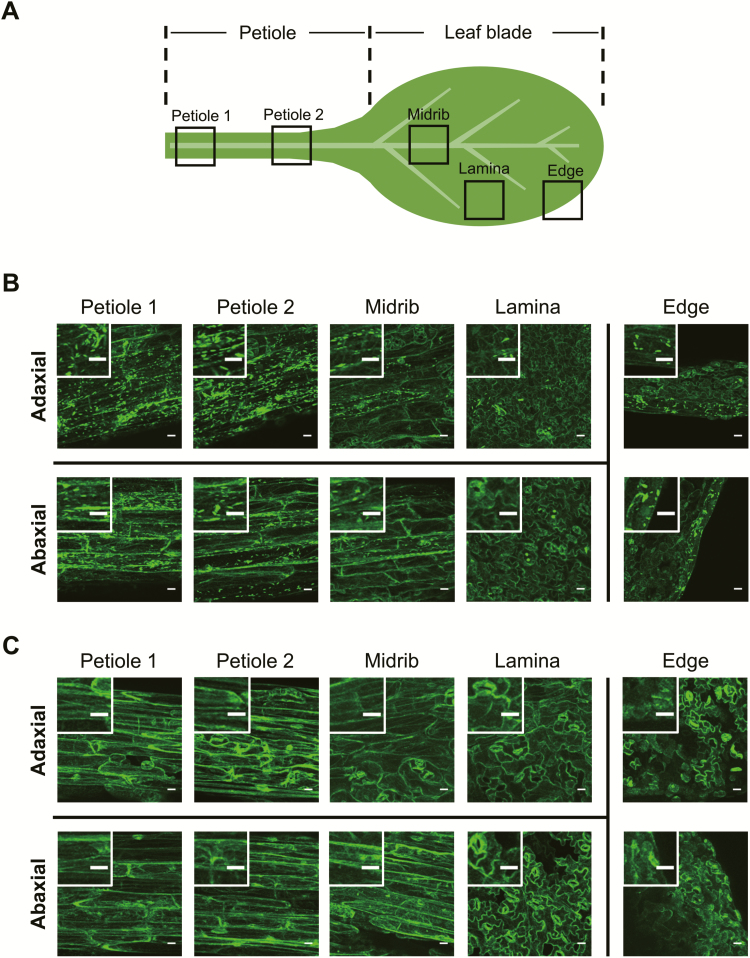
Tissue distribution of ER bodies in Arabidopsis leaves. The first true leaf from a 14-day-old plant grown under normal conditions was used. (A) Leaf portions subjected to fluorescence microscopic observation for GFP. (B and C) Representative fluorescence images of different leaf portions from *GFP-h* (B) and *GFP-h nai2-2* plants (C), with enlarged images in the insets. Scale bars=20 μm.

Interestingly, before transient expression, we observed numerous spindle-shaped GFP spots in the leaf petiole and some in the leaf blade (more in the midrib and edge and fewer in the lamina) of *GFP-h* plants in the WT background (Fig. 2A, B). These ER bodies were evenly distributed on the adaxial and abaxial sides of the leaf. These observations indicate that the formation of these structures was not induced by bombardment but rather existed constitutively. In contrast, few GFP spots were detected in *GFP-h nai2-2* plants (Fig. 2C). Because *nai2* mutants lack constitutive ER bodies (Yamada *et al.*, 2008), these observations indicate that substantial amounts of BGLU18 are localized to previously unrecognized constitutive ER bodies that occur in the leaf petioles of Arabidopsis plants. The predominant distribution of BGLU18 in the leaf petiole suggests that this protein is physiologically important in this part of the leaf. We therefore focused our analysis on leaf petioles.

### ER bodies increase in number in response to abiotic stress and undergo dynamic changes during dehydration stress and recovery

**Fig. 3.**
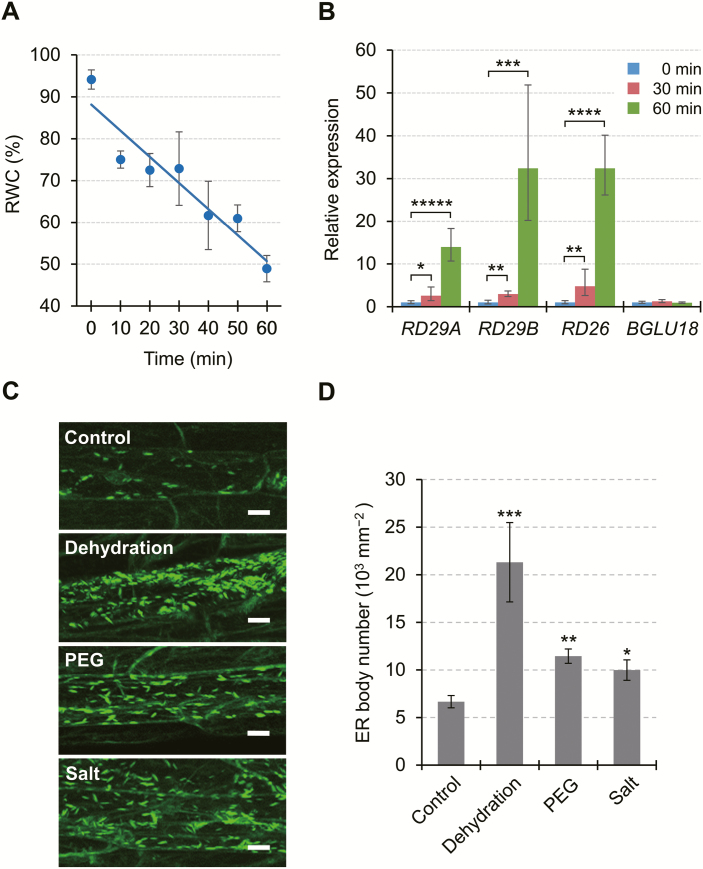
Responses of ER bodies to abiotic stress in epidermal cells of Arabidopsis leaf petioles. (A) Effects of drought-induced dehydration stress on leaf relative water content (RWC). Aseptically grown plants were exposed to dry air for up to 60 min by removing the lids from Petri dishes, and leaves were detached for RWC measurements (*n*=3, where each sample consisted of at least 10 plants). The straight line corresponds to a linear model fitted to the measured data (*y*= –0.6235*x*+88.148, *r*^2^=0.8959, *P*<0.005). (B) Effects of dehydration stress on stress-responsive gene expression. Total RNA was extracted from the aerial parts of dehydration-stressed plants at the indicated time points. Transcript levels of target genes were measured by RT–qPCR, normalized to those of *SAND* as a reference gene, and represented as values relative to the level at the start of stress (0 min), which was given a value of 1. See Supplementary Fig. S5 for the results using other reference genes. PCR primer sequences are provided in Supplementary Table S1. Data are means ±SD (*n*=3), and asterisks denote significant differences between the 0 min value and the 30 or 60 min value in individual genes (*P<0.05; **P<0.005; ***P<0.0005; ****P<0.000005; *****P<0.000001 by Student’s *t*-test). (C) Representative fluorescent images of epidermal cells of *GFP-h* plants subjected to dehydration, PEG-mediated osmotic stress, or high salinity (Salt). Dehydration stress was applied as described for 60 min, while osmotic and salt stress was applied by transferring plants onto solid medium containing PEG (equivalent to −0.5 MPa) or 150 mM NaCl, respectively, and incubating for 12 h. Scale bars=20 μm. (D) Number of ER bodies in leaf petiole epidermal cells of *GFP-h* plants exposed to various stresses. Data are means ±SD (*n*=4; *P<0.05; **P<0.001; ***P<0.00001 by Student’s *t*-test compared with the control).

Since BGLU18 is a key enzyme in ABA production and ABA plays pivotal roles in adaptive responses to abiotic stress, we investigated whether ER bodies in leaf petioles respond to abiotic stress by subjecting *GFP-h* plants to drought-induced dehydration stress, PEG-induced osmotic stress, and high salinity (Fig. 3). For dehydration treatment (up to 60 min), we monitored changes in the dehydration status of stressed plants by measuring leaf RWC and the transcript levels of three canonical stress-responsive genes, *RESPONSIVE TO DESICCATION 29A* (*RD29A*; At5g52310), *RD29B* (At5g52300), and *RD26* (At4g27410), along with *BGLU18*, which is also known to be induced by dehydration (Lee *et al.*, 2006; Supplementary Fig. S4). Leaf RWC decreased progressively (Fig. 3A), whereas the transcript levels of all genes except *BGLU18* significantly increased with increasing duration of dehydration stress (Fig. 3B; Supplementary Fig. S5), thus supporting the validity of the stress treatment. Under dehydration treatment, the number of petiole ER bodies increased significantly (3.2-fold) compared with the unstressed controls (Fig. 3C, D). The number of ER bodies also increased significantly after a 12 h treatment with NaCl or PEG (1.5- to 1.7-fold). These results demonstrate that in the leaf petiole, ER bodies not only exist constitutively, but their formation is also induced by these abiotic stresses.

**Fig. 4.**
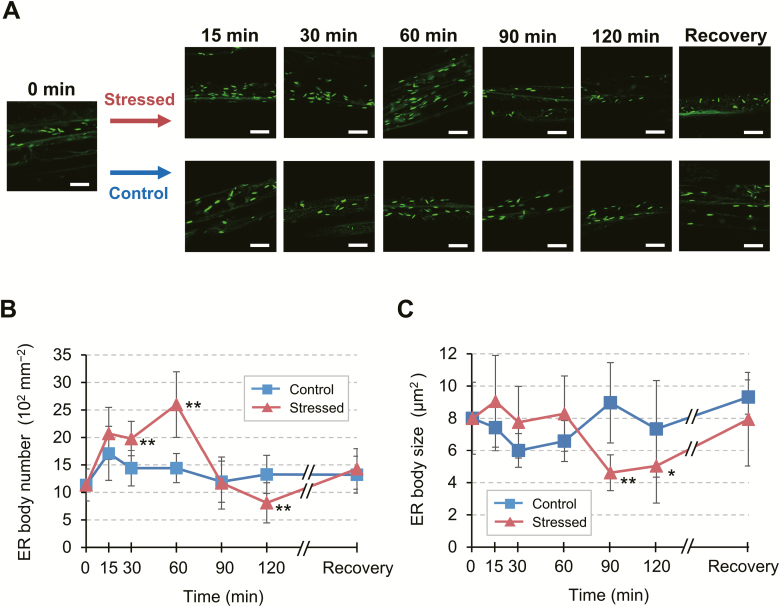
Dynamic changes in leaf petiole ER bodies in response to drought-induced dehydration stress. (A) Representative time-course fluorescence images of leaf petiole epidermal cells of a *GFP-h* plant exposed to dehydration stress. Aseptically grown plants were subjected to dry air for the indicated time period up to 120 min. For recovery, stressed plants were restored to normal conditions and grown for an additional 18 h. Scale bars=20 μm. (B and C) Time-course of changes in the number (B) and average size (C) of ER bodies. Two-dimensional sizes of individual ER bodies, as indicated by green spot areas, were determined as described in the Materials and methods. Data are means ±SD (*n*=4 in B; *n*=5 in C; *P<0.05; **P<0.01 by Student’s *t*-test compared with the control at the same time point).

To examine the responses of leaf petiole ER bodies to abiotic stress in further detail, we monitored the changes in their number and size over the course of a 120 min dehydration treatment and following a period of recovery (Fig. 4). Compared with control conditions, the number of ER bodies began to increase significantly within 30 min after the onset of stress and nearly doubled at 60 min, after which it returned to the original level (90 min) and decreased further (120 min) (Fig. 4A, B). Coincident with the decline in the number of ER bodies, their average size was also reduced at 90 min and 120 min (Fig. 4A, C). However, these ER bodies returned to their original state after recovery from dehydration, suggesting that the observed changes are part of the physiological response to stress treatment. These results demonstrate that the ER in the leaf petiole undergoes dynamic changes, as evidenced by the reversible changes in ER body status, during dehydration stress and recovery.

### The number of ER bodies increases in *aln* mutants and in response to allantoin treatment, causing stress-independent BGLU18 activation, but the *bglu18* mutation abrogates this increase

**Fig. 5.**
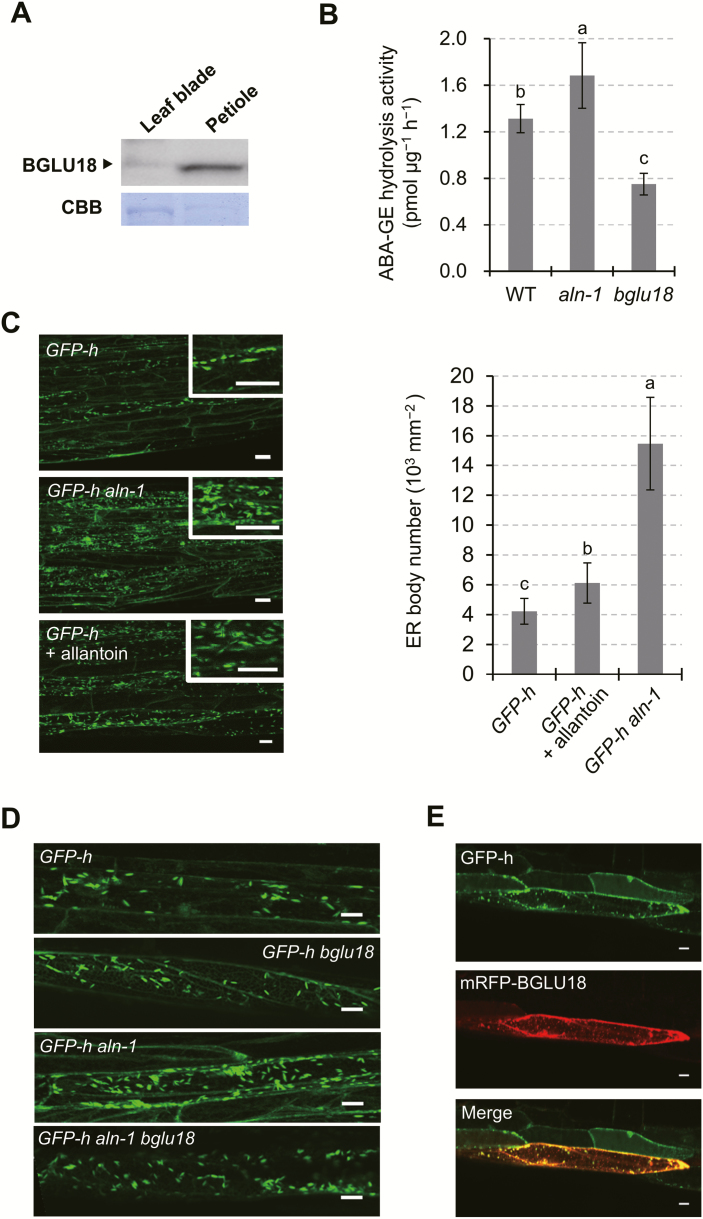
Effects of *aln* mutation and exogenous allantoin on the ABA-GE activity of BGLU18 and ER bodies in leaf petioles. (A) Distribution of BGLU18 in *aln-1* leaf tissue. Immunoblotting was carried out as described in Fig. 1A. (B) ABA-GE hydrolysis activity in WT, *aln-1*, and *bglu18* plants. (C) Representative fluorescence images of leaf petiole epidermal cells in *GFP-h* (upper), *GFP-h aln-1* (middle), and *GFP-h* plants grown in the presence of 100 μM allantoin (lower). The histogram on the right shows the quantity of ER bodies in leaf petiole epidermal cells. (D) Representative fluorescence images of leaf petiole epidermal cells in *GFP-h* (uppermost), *GFP-h bglu18* (upper middle), *GFP-h aln-1* (lower middle), and *GFP-h aln-1 bglu18* plants (lowermost). (E) Representative fluorescence images of leaf petiole epidermal cells transiently expressing *mRFP-BGLU18* in *GFP-h aln-1* plants. Data are means ±SD (*n*=3 in B; *n*=5 in C). Different letters indicate a significant difference (*P*<0.05 by one-way ANOVA). Scale bars=20 μm.

To assess the relationship between BGLU18 and abiotic stress responses of leaf petiole ER bodies, we examined the status of ER bodies in the *aln-1* mutant, which accumulates allantoin, since this purine metabolite activates BGLU18 and increases basal ABA levels under normal conditions (Watanabe *et al.*, 2014*b*). In the *aln-1* mutant, like WT plants, BGLU18 predominantly localized to leaf petioles (Fig. 5A). Consistent with previous findings, ABA-GE hydrolysis activity was highest in *aln-1*, followed by the WT, and lowest in *bglu18*, which we used as a background control given that BGLU18 is a member of a large enzyme family (47 members in Arabidopsis; Xu *et al.*, 2004; Nakano *et al.*, 2014) (Fig. 5B). We crossed *aln-1* with the *GFP-h* line to visualize the ER and ER bodies. In the absence of stress, the resulting *GFP-h aln-1* plants had significantly (3.8-fold) more ER bodies in petiole tissues than *GFP-h* plants (Fig. 5C). Treating the parental *GFP-h* plants with exogenous allantoin (100 μM) resulted in a similar increase in the number of ER bodies, confirming the notion that allantoin increases the abundance of ER bodies. However, introducing the *bglu18* mutation into the *GFP-h aln-1* mutant background (*GFP-h aln-1 bglu18*) decreased ER body number to normal levels, as observed in *GFP-h* and *GFP-h bglu18* plants (Fig. 5D). These findings suggest that BGLU18 is a necessary component in the induction of ER body formation in the leaf petiole. This idea is supported by the observation that transient expression of *mRFP-BGLU18* in the *GFP-h aln-1* mutant resulted in strong RFP fluorescence on ER bodies (Fig. 5E). Collectively, these results uncover a direct relationship between ER body number and BGLU18-mediated ABA-GE hydrolysis activity.

### BGLU18 remains in the ER and enhances ABA-GE hydrolysis activity to increase ABA levels under dehydration stress

ER bodies are subcellular compartments that function in the temporary storage of certain BGLUs, such as BGLU23, which are released from the ER bodies upon stress to react with substrates stored separately in other compartments, such as vacuoles (Yamada *et al.*, 2011; Nakano *et al.*, 2014). Whether the BGLU18 substrate, ABA-GE, is stored in the ER is unclear, although it forms in the cytosol and is transported into the vacuole for intracellular storage (Harris and Dugger, 1986; Burla *et al.*, 2013; Dong *et al.*, 2014).

**Fig. 6.**
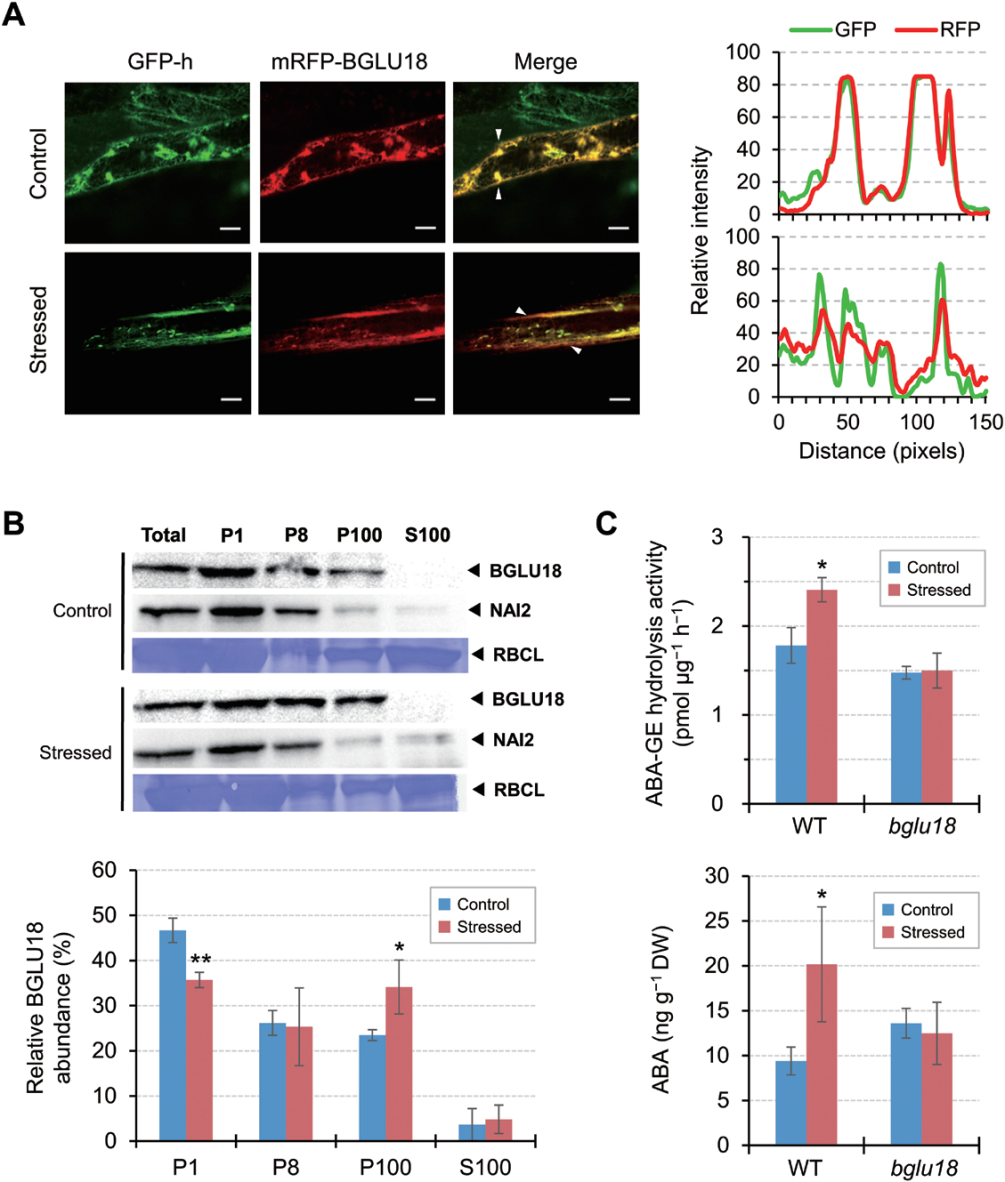
Effects of drought-induced dehydration on the subcellular distribution and ABA-GE hydrolysis activity of BGLU18 and ABA levels in leaf petioles. (A) Left: representative fluorescence images of leaf petiole epidermal cells transiently expressing *mRFP-BGLU18* in *GFP-h* plants under control or stress conditions (as in Fig. 4 for 30 min). Scale bars=20 μm. Right: line profile graphs showing relative pixel intensities for GFP (green) and mRFP (red) fluorescence (upper, control; lower, stressed), which were line-scanned and quantified between the two white arrowheads in the merged panels on the left using ImageJ. (B) Subcellular distribution of BGLU18. Biochemical fractionation followed by immunoblotting was performed starting with control plants and plants exposed to dehydration for 30 min. Bar graphs below show the relative distribution of BGLU18 in the four fractions. (C) ABA-GE hydrolysis activity (upper) and ABA levels (lower) in WT and *bglu18* plants under control conditions or exposed to dehydration for 30 min. Leaf petioles were collected from control and dehydration-stressed plants of each genotype and assayed for ABA by LC-ESI-MS/MS on a DW basis. Data are means ±SD (*n*=3 in both B and C). Significant differences between treatments are indicated by asterisks (*P<0.05; **P<0.005 by Student’s *t*-test).

Therefore, we investigated whether abiotic stress affects the subcellular localization of BGLU18. When we exposed *GFP-h* plants transiently expressing *mRFP-BGLU18* to a 30 min dehydration stress (Fig. 4), the ER status of the stressed cells was significantly altered compared with that of control cells (Fig. 6A, left of the left panel). Under stress conditions, mRFP signals, which overlapped fully with GFP signals under control conditions, had slightly diffused from major GFP spots (ER bodies) and became a bit more evenly distributed within the cell (Fig. 6A, middle and right of the left panel). This observation was supported by quantitative analysis of relative fluorescence intensities across the cell (Fig. 6A, right panel). We also examined the distribution of endogenous BGLU18 in subcellular fractions obtained from dehydration-stressed plants by immunodetection. Dehydration treatment increased the level of BGLU18 in the microsomal P100 fraction, albeit to a small extent, suggesting that stress affects the relative distribution of BGLU18 between ER bodies and microsomes (consisting of ER membranes and lumen proteins) (Fig. 6B). However, the protein was barely detected in the S100 fraction, indicating that BGLU18 primarily remains in the ER and ER bodies under dehydration stress.

We investigated whether these changes in distribution occurred in conjunction with changes in the ABA-GE hydrolysis activity of BGLU18 and with ABA levels in the leaf petiole. BGLU18 activity significantly increased in WT plants exposed to dehydration compared with non-stressed plants, whereas BGLU18 activity in *bglu18* was similar under both conditions (Fig. 6C). ABA-GE hydrolysis activity in the WT was estimated to increase 3-fold when background *bglu18* activity was subtracted from each condition. Along with increasing enzyme activity, ABA levels significantly increased (2-fold) in leaf petioles from stressed plants. Overall, these results uncover a link between stress-induced ER dynamics, BGLU18 activation, and ABA levels.

### Loss of constitutive ER bodies enhances BGLU18 activity under normal and dehydrated conditions, but ABA levels increase only under dehydrated conditions

**Fig. 7.**
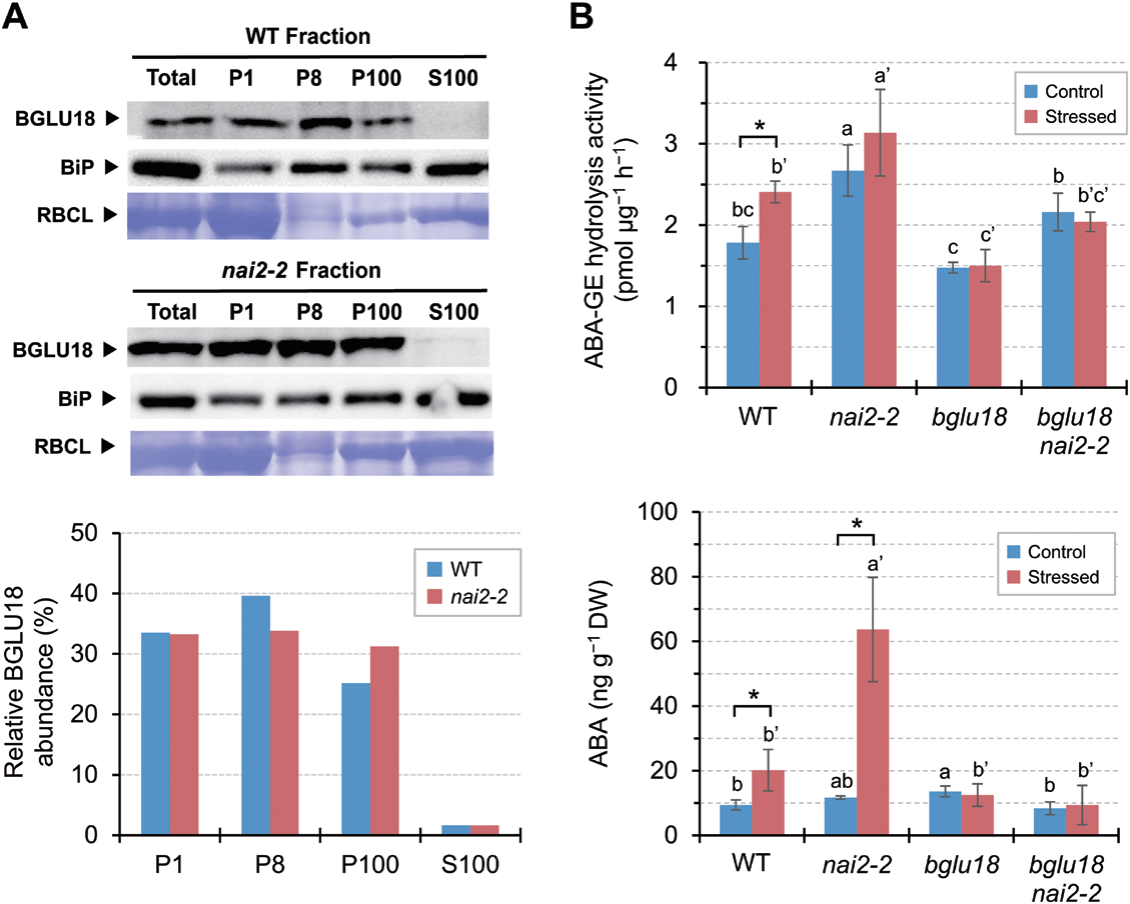
Effects of *nai2* mutation on the subcellular distribution and ABA-GE activity of BGLU18 and on ABA levels in leaf petioles. (A) Subcellular distribution of BGLU18. Biochemical fractionation followed by immunoblotting analysis were performed as described in Fig. 1B. Bar graphs below show the relative distribution of BGLU18 in the four fractions. The experiments were repeated twice with similar results; results from one experiment are shown. (B) ABA-GE hydrolysis activity (upper) and ABA levels (lower) in *nai2-2* single and *nai2-2 bglu18* double mutant plants under control conditions or exposed to dehydration for 30 min. WT and *bglu18* data used for comparison are from Fig. 6C, as the experiments were done in parallel. Leaf petioles were collected from control and dehydration-stressed plants and assayed for ABA by LC-ESI-MS/MS on a DW basis. Data are means ±SD (*n*=3). Significant differences between treatments are indicated by asterisks (*P<0.05 by Student’s *t*-test) and those among genotypes by different letters (*P*<0.05 by one-way ANOVA).

To further investigate the causal relationship between the changes in BGLU18 distribution in the ER system and increased BGLU18 activity and ABA levels under dehydration stress, we examined the *nai2-2* mutant, which is deficient in constitutive ER bodies. Since this mutation has little or no effect on BGLU18 protein levels (Supplementary Fig. S6), we predicted that the loss of constitutive ER bodies, which accumulate BGLU18 (Fig. 1), would result in the increased distribution of this enzyme to microsomes under normal conditions. Consistent with the results described above (Fig. 6B), WT plants contained more BGLU18 in the ER-body-rich P1 and P8 fractions than in the microsomal P100 fraction (Fig. 7A). In contrast, in *nai2-2*, BGLU18 was distributed nearly evenly between the three fractions. These results indicate that the relatively high levels of BGLU18 in the microsomes of *nai2-2* are due to its inability to form constitutive ER bodies.

We measured ABA-GE hydrolysis activity and leaf petiole ABA levels in *nai2-2*, along with the *bglu18 nai2-2* double mutant as a background control (Supplementary Fig. S7), and compared these results with those for the WT and *bglu18* single mutant (Fig. 7B). The *nai2-2* plants showed the highest and the *bglu18* plants the lowest ABA-GE hydrolysis activity under both normal and stress conditions (Fig. 7B, upper). Introducing the *bglu18* mutation into the *nai2-2* background resulted in lower enzyme activity in the *bglu18 nai2-2* double mutant compared with *nai2-2* single mutant plants, suggesting that the *nai2-2* mutation caused increased activity of BGLU18 and possibly other enzymes capable of degrading ABA-GE. Along with enzyme activity, dehydration stimulated ABA accumulation by 5-fold in *nai2-2* (Fig. 7B, lower) to a level significantly higher than that in WT (2-fold increase). Under normal conditions, however, this increased enzyme activity did not lead to increased ABA levels in *nai2-2*. Neither the *bglu18* single nor the *bglu18 nai2-2* double mutant responded to dehydration stress treatment by increasing ABA levels, suggesting that *de novo* synthesis contributed little to increased ABA levels under our experimental conditions, as examined further below. These results indicate that dehydration-responsive ABA production occurs in leaf petioles, a process mediated by BGLU18 and augmented by the loss of constitutive ER bodies, but this occurs only under stress conditions.

### Loss of BGLU18 causes a delay in dehydration-induced ABA accumulation

**Fig. 8.**
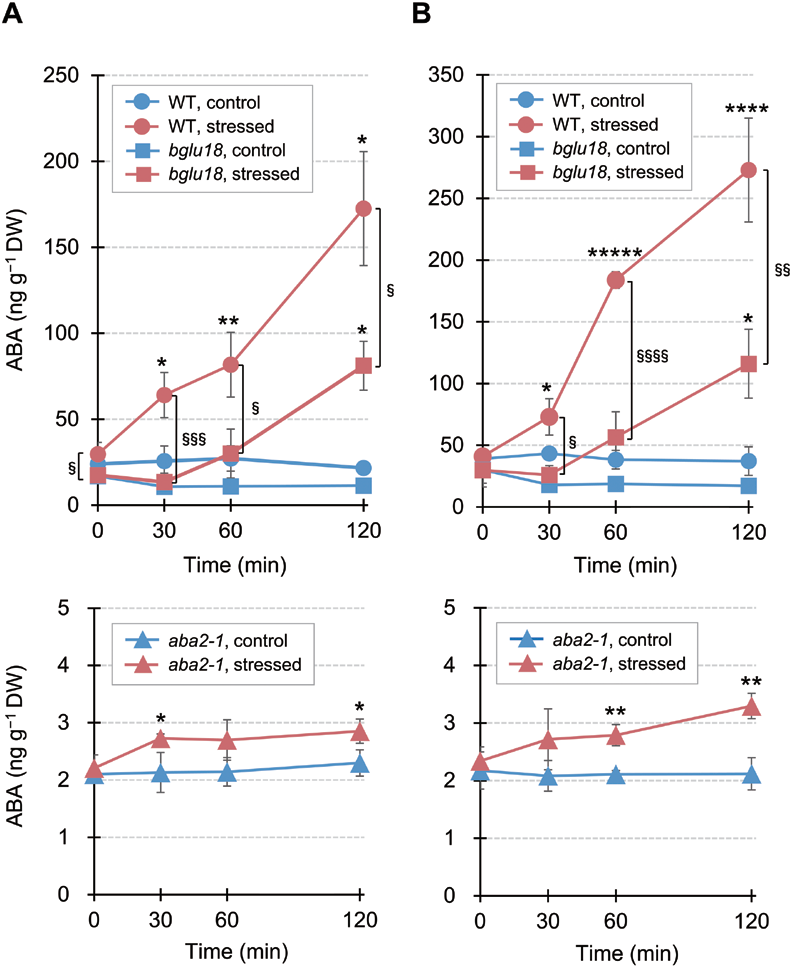
Effects of *bglu18* and *aba2-1* mutations on time-course changes in dehydration-induced ABA accumulation in leaf petioles and blades. Aseptically grown plants were subjected to dry air for the indicated time periods up to 120 min, and leaf petioles (A) and blades (B) were separately collected for ABA measurements by LC-ESI-MS/MS on a DW basis. Data are means ±SD (*n*=3). Significant differences between treatments are indicated by asterisks (*P<0.05; **P<0.01; ***P<0.005; ****P<0.001; *****P<0.00005 by Student’s *t*-test) and those between genotypes by section marks (^§^*P*<0.05; ^§§^*P*<0.01; ^§§§^*P*<0.005; ^§§§§^*P*<0.001 by Student’s *t*-test).

To examine the temporal and spatial contribution of BGLU18 to the early stage of stress-induced ABA accumulation, we monitored changes in ABA levels in the leaf tissue of WT plants and two ABA metabolism mutants, *bglu18* and *aba2-1*, during a 120 min dehydration treatment (Fig. 8). ABA levels in WT petioles significantly increased (>2-fold) within 30 min of the onset of stress treatment and continued to increase (5.8-fold) by 120 min of treatment, whereas they remained steady under control conditions (Fig. 8A, upper panel). In dehydration-stressed *bglu18* petioles, the ABA levels did not increase significantly until 60 min (*P*>0.3 by one-way ANOVA) and then increased 4.6-fold at 120 min, revealing the delayed increase in ABA accumulation in the absence of BGLU18. ABA levels in *aba2-1*, which is impaired in *de novo* ABA biosynthesis, were only 8–11% and 12–20% those of the WT and *bglu18*, respectively, under control conditions. However, ABA levels increased slightly but significantly (1.2-fold) within the first 30 min of dehydration treatment (Fig. 8A, lower panel), suggesting that ABA-GE hydrolysis contributes to the early response to dehydration stress, which is probably mediated by BGLU18. ABA levels in leaf blades of the three genotypes exhibited a similar pattern to those in the petiole under both control and stress conditions, with slightly higher levels in leaf blades versus petioles (Fig. 8B). Taken together, these results indicate that BGLU18 is involved in an early stage of ABA accumulation not only in the petioles but also in the blades of dehydration-stressed leaves.

## Discussion

The regulation of ABA accumulation is of fundamental importance for plant responses to abiotic stresses such as drought. Although roots are the primary sites for sensing drought, there is evidence that the foliar production of ABA is independently required to elicit stress responses in leaves (Holbrook *et al.*, 2002; Christmann *et al.*, 2007). However, in contrast to the extensive research on the multistep process of *de novo* ABA biosynthesis, far fewer studies have investigated the single-step hydrolysis of ABA-GE, which allows for quick ABA production. To explore the cellular mechanism for the activation of foliar ABA production, we focused on BGLU18, a key enzyme for ABA-GE hydrolysis, in drought-stressed Arabidopsis leaves (Lee *et al.*, 2006; Watanabe *et al.*, 2014*b*). Our results highlight the involvement of stress-induced ER dynamics in BGLU18-mediated ABA production, which has intriguing implications for the regulation of cellular ABA homeostasis during stress responses and adaptation. Our results also shed light on the physiological functions of ER bodies, which remain to be fully explored.

We first addressed the localization of BGLU18 in leaves at the tissue and subcellular levels. BGLU18 was originally reported to be localized to the ER of leaf protoplasts based on a constitutive overexpression study (Lee *et al.*, 2006). However, the enzyme was subsequently identified as a major component of ER bodies whose formation was induced in wounded cotyledons (Ogasawara *et al.*, 2009). ER bodies that are constitutively present but limited to specific epidermal cells of leaf blades were recently identified and contained BGLU18 (Nakazaki *et al.*, 2019). Here, we showed that this enzyme is present at higher levels in leaf petioles than in leaf blades, where it is predominantly, but not exclusively, localized to the ER bodies of epidermal cells (Figs 1, 2). In addition, the current findings, together with those of Nakazaki *et al.* (2019), establish that ER bodies are constitutively present in true leaves of Arabidopsis plants, even though it was previously thought that they rarely occur in healthy rosette leaves (Matsushima *et al.*, 2002; Nakano *et al.*, 2014).

ER bodies increase in number in response to mechanical wounding in Arabidopsis cotyledons and leaves (Matsushima *et al.*, 2002; Ogasawara *et al.*, 2009). Wound-inducible ER bodies, together with constitutive ER bodies, probably play a defensive role against biotic stresses such as herbivory and pathogenesis (Sherameti *et al.*, 2008; Yamada *et al.*, 2011; Nakano *et al.*, 2017; Nakazaki *et al.*, 2019). Due to the known role of BGLU18 in ABA metabolism, we investigated whether ER bodies in leaves respond to abiotic stress conditions, which had not been examined previously. In a pattern similar to the wounding response, the number of ER bodies in leaf petioles increased significantly in response to drought-induced dehydration stress, osmotic stress, or high salinity (Fig. 3). Thus, dynamic changes in ER body status constitute a general stress response in Arabidopsis. Nevertheless, the changes in leaf petioles occurred over a much shorter period (30–60 min for dehydration and 12 h for osmotic and salt stress; Figs 3, 4) than the time required for wounding responses in leaf blades (44–66 h; Matsushima *et al.*, 2002). In addition, the dehydration response of ER bodies in stressed cells was reversed following the removal of stress (Fig. 4), whereas the wounding response involving ER bodies requires cellular destruction to exert a so-called ‘mustard oil bomb’ strategy, producing secondary metabolites toxic to potential pathogens and herbivores (Yamada *et al.*, 2011; Nakano *et al.*, 2017). These differences might reflect the distinct roles played by ER bodies whose formation is induced upon dehydration and wounding, that is, in abiotic versus biotic stress responses.

Given our observations in leaf petiole tissues, we were interested in exploring whether abiotic stress-induced dynamic changes in ER body status were physiologically relevant to BGLU18 function. We examined the effect of allantoin on ER body dynamics, as this ER-related purine metabolite activates BGLU18 and enhances basal ABA levels, while the inability to produce allantoin results in hypersensitivity to drought in Arabidopsis (Watanabe *et al.*, 2010, 2014*a*, *b*). Both endogenously accumulated (resulting from the *aln* mutation) and exogenously applied allantoin caused an increase in leaf petiole ER bodies in the absence of stress (Fig. 5), providing a link between the dynamic behavior of ER bodies and BGLU18 activation. How allantoin induces such ER dynamics remains to be elucidated.

We further explored the relevance of ER dynamics to BGLU18 activation, as ER bodies were previously considered to be physiologically inert (Herman and Schmidt, 2004; Yamada *et al.*, 2011; Nakano *et al.*, 2014). In contrast to NAI2, an ER body-specific protein (Yamada *et al.*, 2008), BGLU18 was not exclusively localized to ER bodies; a certain amount was detected in the microsomal (ER) fractions under normal conditions (Fig. 1B). Intriguingly, drought-induced dehydration stress not only triggered changes in ER body status but also led to a relative increase in BGLU18 levels in microsomes, resulting in enhanced ABA-GE hydrolysis activity and increased ABA concentrations (Fig. 6). In contrast, under our experimental conditions, neither enzyme activity nor ABA levels were altered in the *bglu18* mutant exposed to the same stress treatment. These results suggest that stress-induced ER body dynamics somehow enhance BGLU18-mediated ABA production by affecting the relative distribution of the enzyme between the ER and ER bodies. The mechanisms causing such sub-ER distributional changes in BGLU18 are currently unknown. Notably, upon exposure to dehydration, the size of ER bodies decreased following a transient increase in their numbers (Fig. 4).

Perhaps ER bodies undergo partial degradation or disorganization to liberate BGLU18 into the ER, where catalysis might occur. We tested this possibility using an ER body-deficient *nai2-2* mutant. We reasoned that BGLU18 would reside in the ER if the formation of constitutive ER bodies was prevented by the *nai2-2* mutation, as the mutation did not affect BGLU18 protein levels. Compared with the WT, BGLU18 was relatively enriched in microsomes in the *nai2-2* mutant, where ABA-GE hydrolysis activity was significantly higher under both normal and stress conditions (Fig. 7B). These results suggest that the disorganization of the ER body represents a key process for BGLU18 activation. Despite the increased enzyme activity, however, this mutant overaccumulated ABA only when exposed to stress (to a level >3-fold higher than that of the equally stressed WT; Fig. 7B). Similarly, Lee *et al.* (2006) demonstrated that constitutive BG1/BGLU18 overexpression in Arabidopsis profoundly increased foliar ABA levels under dehydration stress but not control conditions. Our results support the idea that ABA-GE is not normally stored in the ER but is transported there from the apoplast and/or vacuoles via an as yet unknown stress-stimulated mechanism (Dietz *et al.*, 2000; Lee *et al.*, 2006). Alternatively, as ER bodies function in ER to vacuole trafficking pathways (Hayashi *et al.*, 2001; Herman and Schmidt, 2004), it is conceivable that upon stress exposure, BGLU18 is transported to the vacuole, where it hydrolyzes ABA-GE. However, this is unlikely given our findings on mRFP–BGLU18 localization under dehydration stress (Fig. 6A).

An obvious advantage of ABA production from ABA-GE lies in its status as a single-step reaction, which occurs much more rapidly than the multistep process of ABA biosynthesis. Thus, BGLU18 is thought to enable the rapid production of ABA in order to increase cellular levels locally rather than overall (Lee *et al.*, 2006). Our finding that BGLU18 activation occurs shortly (30 min) after the onset of dehydration stress (Figs 6, 7) is consistent with this hypothetical role of BGLU18, and hence ABA-GE hydrolysis, in stress responses. This idea was further supported by our time-course comparison of dehydration-induced ABA accumulation in leaves among genotypes, as the *bglu18* mutant did not exhibit the early ABA accumulation that was observed in the WT (Fig. 8). Conversely, the *aba2-1* mutant showed slightly but significantly earlier ABA production in response to dehydration, even though *de novo* ABA synthesis is largely impaired in this mutant. This small but early increase probably results from the deconjugation of ABA-GE. These results strongly suggest that the rapid activation of BGLU18 is responsible for the early ABA accumulation that precedes *de novo* biosynthesis, which might occur via a mechanism involving ER dynamics at the organellar level (this study) and post-translational regulation at the molecular level (Lee *et al.*, 2006; Watanabe *et al.*, 2014*b*); however, the transcriptional activation of *BGLU18* is unlikely based on RT–qPCR (Fig. 3B; Supplementary Fig. S5). The time-course ABA analysis also revealed that BGLU18-mediated ABA-GE hydrolysis plays substantial roles in both the petioles and blades of stressed leaves.

The physiological significance of ABA production in the epidermal cells of leaf petioles remains to be addressed. Our results suggest that ABA-GE hydrolysis plays a part in an early stress response to generate ABA, which might contribute to activation of the ABA biosynthetic pathway through positive feedback regulation (Xiong *et al.*, 2002). Such feedback regulation would involve the translocation of ABA from BGLU18-containing epidermal cells to vascular parenchyma cells, where the genes for ABA biosynthetic enzymes are primarily expressed (Koiwai *et al.*, 2004; Endo *et al.*, 2008; Kuromori *et al.*, 2014). The thin, stalk-like structure of the leaf petiole may be favorable for the translocation of ABA since epidermal tissues surround the vascular tissues in closer proximity in petioles compared with leaf blades.

BG1/BGLU18 was originally identified as an ER enzyme involved in ABA homeostasis and metabolism (Lee *et al.*, 2006). However, this enzyme was recently proposed to act as a myrosinase to produce defense compounds from glucosinolate substrates in response to herbivory (Nakazaki *et al.*, 2019). Given that ER bodies in leaves respond to both abiotic and biotic stress (Matsushima *et al.*, 2002; this work), it is likely that BGLU18 plays a dual role, depending on which environmental stress the plant encounters. Since BGLU18 is physically separated from its substrates (i.e. ABA-GE and indole glucosinolates) under normal conditions, the two distinct activities are probably regulated by the physiological process by which each substrate becomes available under stress conditions: as noted above, dehydration-induced ABA production might involve stress-activated transport of ABA-GE into the ER from the apoplast or vacuoles (Lee *et al.*, 2006; this work), whereas wounding-induced production of toxic compounds is achieved via the direct access of the enzyme to vacuole-resident glucosinolate upon cell collapse (Yamada *et al.*, 2011; Nakano *et al.*, 2017). If this scenario is accurate, BGLU18 in leaf petioles might also be involved in defense against biotic stress, as it is in leaf blades.

## Supplementary data

Supplementary data are available at *JXB* online.

Fig. S1. Construction of the *mRFP-BGLU18* fusion plasmid.

Fig. S2. Expression profile of *BG1*/*BGLU18* (At1g52400) in different tissues of Arabidopsis plants during growth.

Fig. S3. Quantitative comparison of *BG1*/*BGLU18* (At1g52400) expression in different tissues of Arabidopsis plants during growth.

Fig. S4. Expression profile of *BG1*/*BGLU18* (At1g52400) in Arabidopsis plants under various abiotic stress conditions.

Fig. S5. Changes in the relative transcript levels of stress-responsive genes during the course of drought-induced dehydration.

Fig. S6. BGLU18 protein levels in WT, *nai2-2*, and *bglu18* plants.

Fig. S7. Genotyping and phenotyping of the *bglu18 nai2-2* double mutant.

Table S1. Primers used in this study.

Table S2. Mass spectrometry settings used for LC-ESI-MS/MS analysis of ABA in negative mode.

erz528_suppl_Supplementary_Data

## Abbreviations

ABA: abscisic acid
ABA-GE: ABA glucosyl ester
aln: *allantoinase*
BG and BGLU: β-glucosidase
BiP: binding protein
ER: endoplasmic reticulum
GFP: green fluorescent protein
GFP-h: GFP with an ER retention signal
mRFP: monomeric red fluorescent protein
PEG: polyethylene glycol
RT–qPCR: reverse transcription–quantitative PCR
WT: wild type
