# Supplementary material for: Dynamics of the leaf endoplasmic reticulum modulate β-glucosidase-mediated stress-activated ABA production from its glucosyl ester
Source: J Exp Bot. 2019 Nov 25;71(6):2058–71. doi: 10.1093/jxb/erz528 (PMC7094080; doi:10.1093/jxb/erz528)
Supplement: erz528_suppl_Supplementary_Data [file erz528_suppl_supplementary_data.pdf]

Supplementary Data: Han *et al*. 10.1093/jxb/erz528

Dynamics of the leaf endoplasmic reticulum modulate ß-glucosidase-mediated stress-activated ABA production from its glucosyl ester

Yiping Han[1], Shunsuke Watanabe[2], Hiroshi Shimada[1,3], Atsushi Sakamoto[1,3,*]

[1]*Graduate School of Science, Hiroshima University, 1-3-1 Kagamiyama, Higashi-Hiroshima 739-8526, Japan*

[2]*RIKEN Center for Sustainable Resource Science, 1-7-22 Suehiro-cho, Tsurumi-ku, Yokohama 230-0045, Japan*

[3]*Graduate School of Integrated Sciences for Life, Hiroshima University, 1-3-1 Kagamiyama, Higashi-Hiroshima 739-8526, Japan*

*Corresponding author: E-mail, ahkkao@hiroshima-u.ac.jp; Fax: +81(0)82 424 4530

The following supplementary data are available for this article:

Figure S1. Construction of the *mRFP-BGLU18* fusion plasmid.

Figure S2. Expression profile of *BG1*/*BGLU18* (At1g52400) in different tissues of Arabidopsis plants during growth.

Figure S3. Quantitative comparison of *BG1*/*BGLU18* (At1g52400) expression in different tissues of Arabidopsis plants during growth.

Figure S4. Expression profile of *BG1*/*BGLU18* (At1g52400) in Arabidopsis plants under various abiotic stress conditions.

Figure S5. Changes in the relative transcript levels of stress-responsive genes during the course of drought-induced dehydration.

Figure S6. BGLU18 protein levels in WT, *nai2-2*, and *bglu18* plants.

Figure S7. Genotyping and phenotyping of the *bglu18 nai2-2* double mutant.

Table S1. Primers used in this study.

Table S2. Mass spectrometry settings used for LC-ESI-MS/MS analysis of ABA in negative mode.

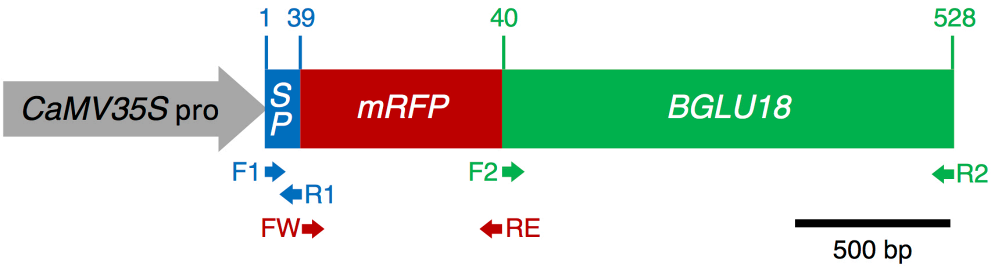

Figure S1. Construction of the *mRFP-BGLU18* fusion plasmid. The coding sequences of the NH_2-terminal region containing a putative signal peptide (SP; residues 1–39) and the mature polypeptide region (residues 40–528) of BGLU18 were individually obtained by PCR using the full-length cDNA clone (GenBank: AY056415) and translationally fused to the 5′ and 3′ ends, respectively, of *mRFP* under the control of the *CaMV35S* promoter in pUGW2 (Nakagawa *et al*., 2007). Arrows denote PCR primers used to construct the plasmid. Primer sequences are shown in Supplementary Table S1.

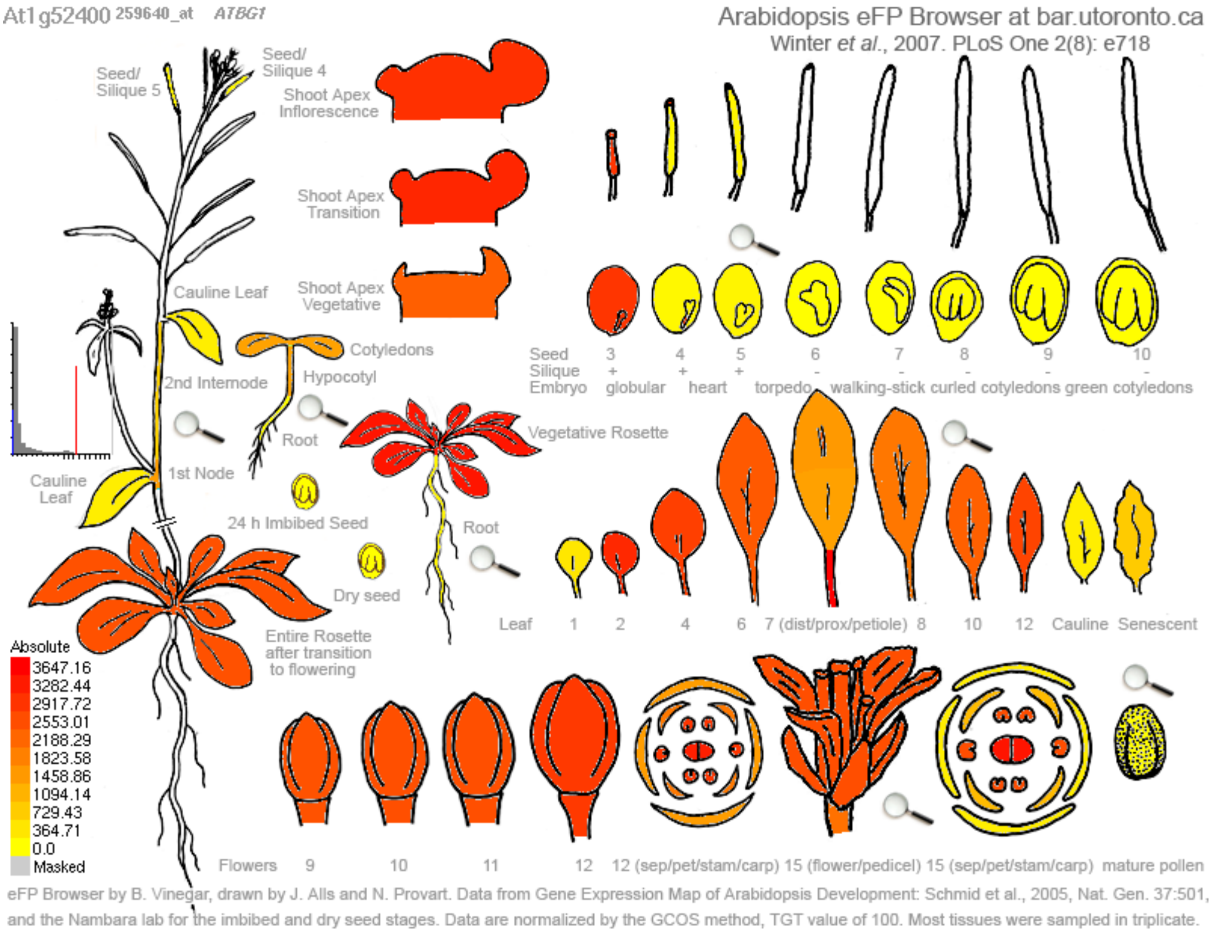

Figure S2. Expression profile of *BG1/BGLU18* (At1g52400) in different tissues of Arabidopsis plants during growth. Obtained from the Arabidopsis eFP browser web server (http://bar.utoronto.ca/efp/cgi-bin/efpWeb.cgi) and reprinted from Winter *et al.* (2007).

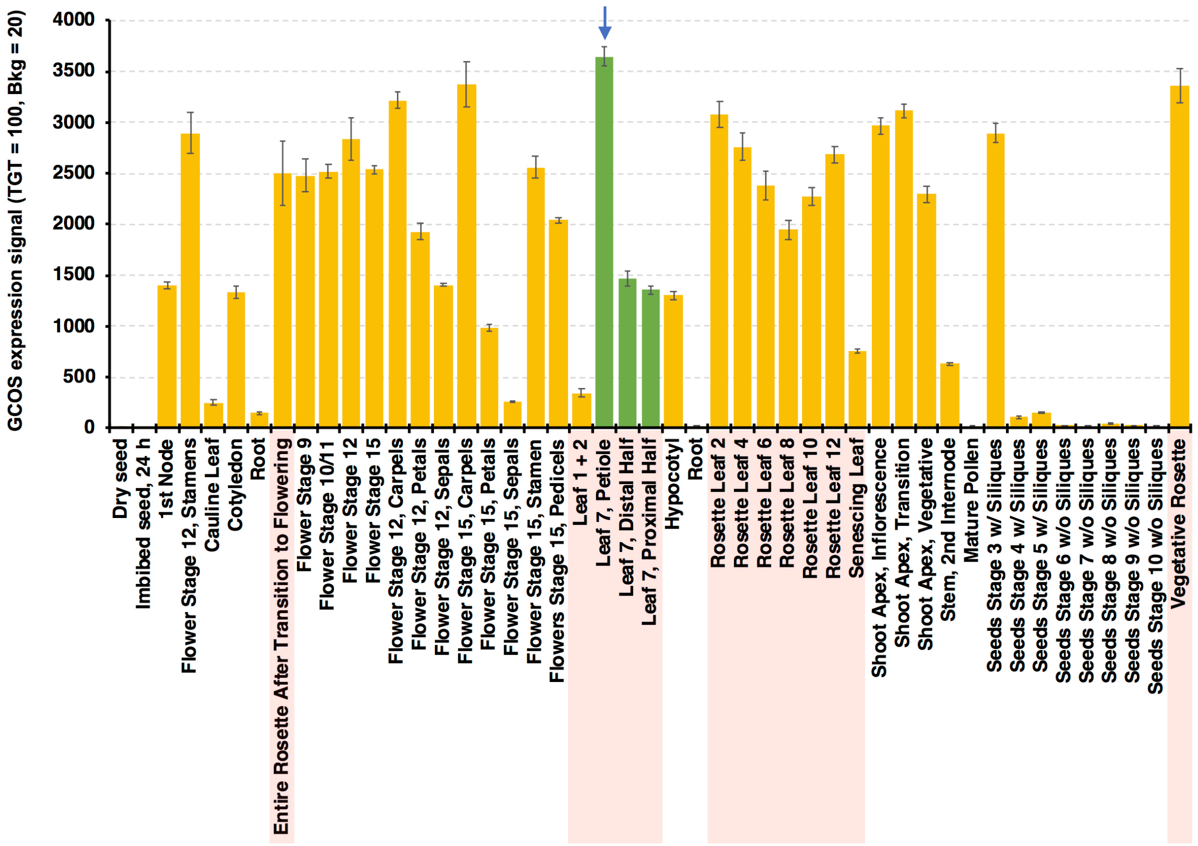

Figure S3. Quantitative comparison of *BG1/BGLU18* (At1g52400) expression in different tissues of Arabidopsis plants during growth. Expression signal values (mean ± SD) were obtained from the Arabidopsis eFP browser web server (http://bar.utoronto.ca/efp/cgi-bin/efpWeb.cgi). Rosette leaf samples are indicated by a pink background. The expression data for three parts (petiole, proximal blade, and distal blade) of a single leaf (rosette leaf 7) are shown in green bars; the expression level in the leaf petiole is indicated by a blue vertical arrow.

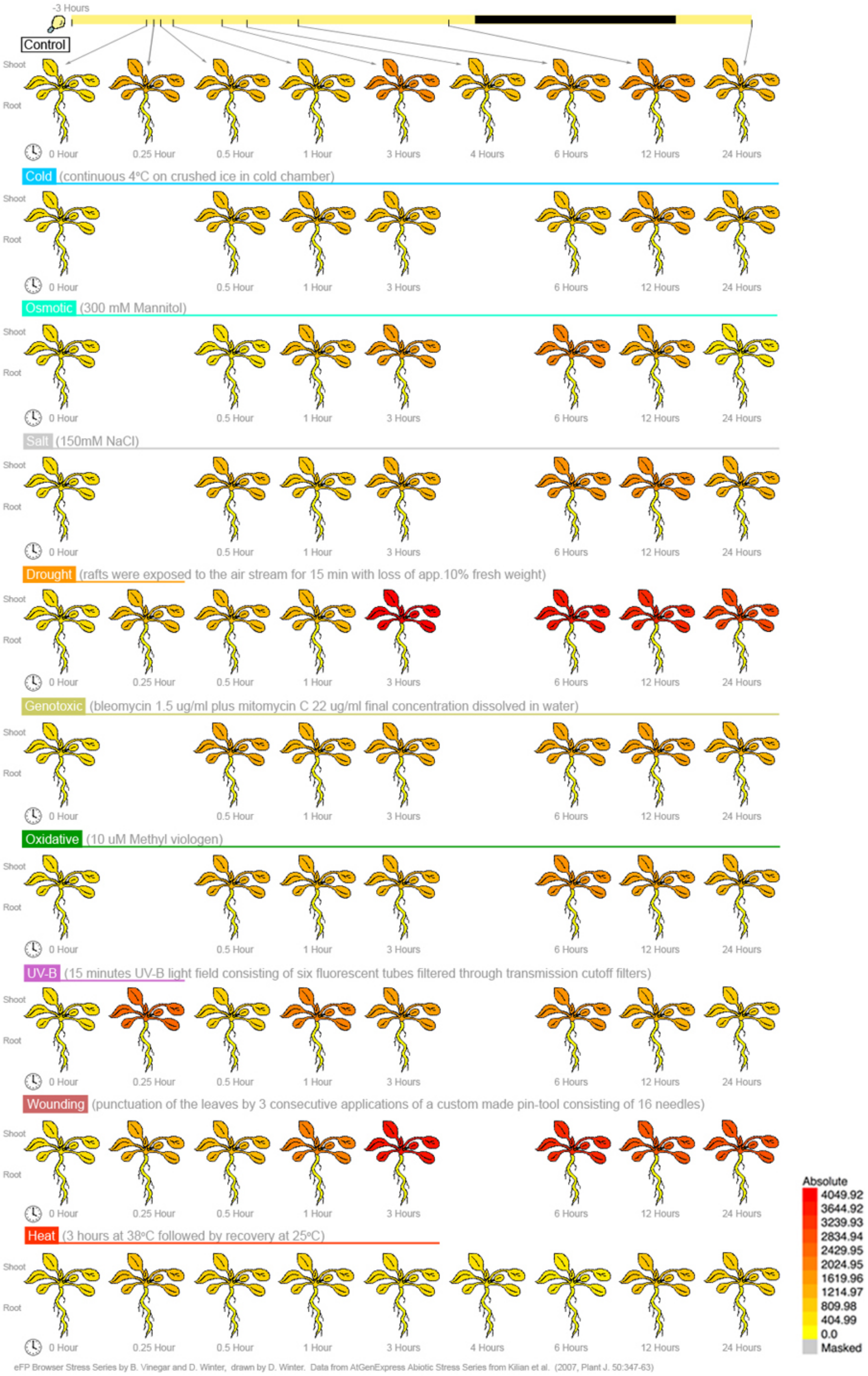

Figure S4. Expression profile of *BG1/BGLU18* (At1g52400) in Arabidopsis plants under various abiotic stress conditions. Obtained from the Arabidopsis eFP browser web server (http://bar.utoronto.ca/efp/cgi-bin/efpWeb.cgi) and reprinted from Winter *et al.* (2007).

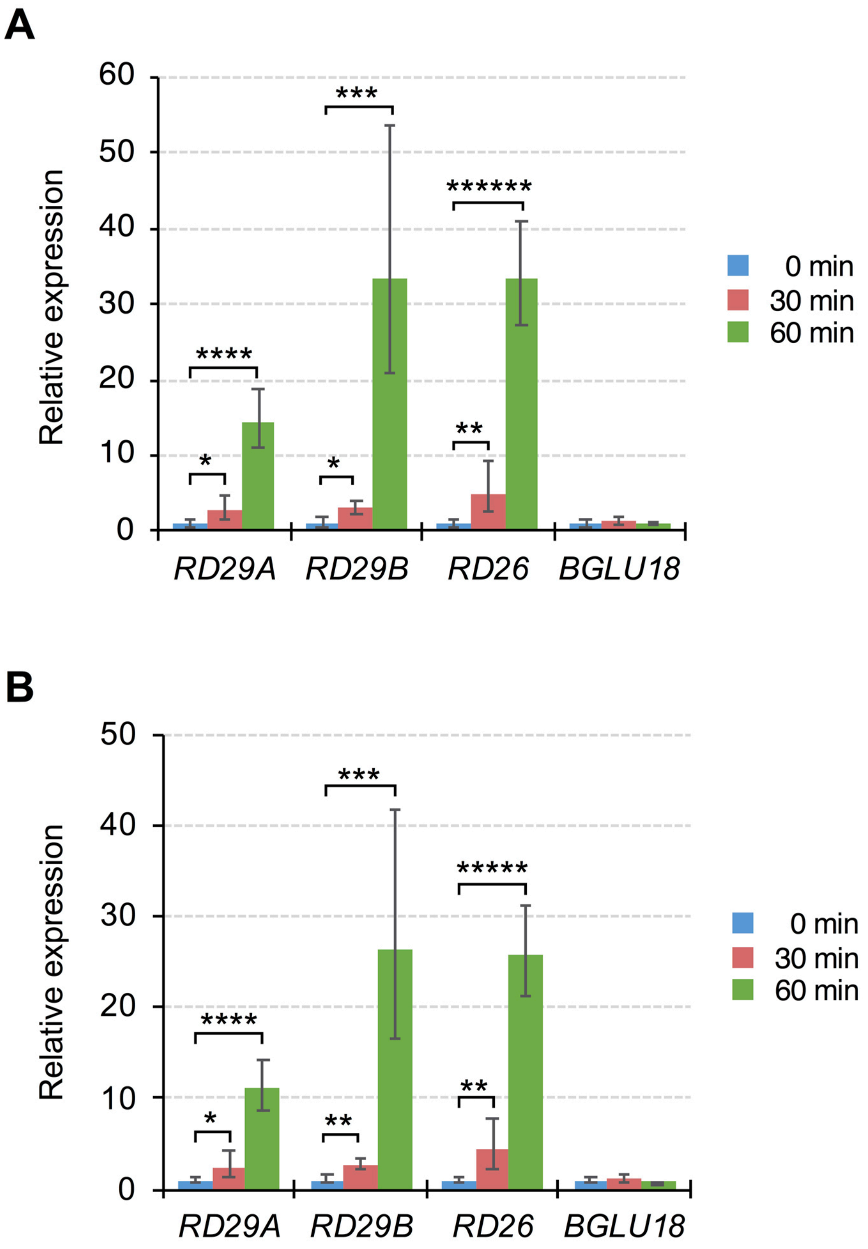

Figure S5. Changes in the relative transcript levels of stress-responsive genes during the course of drought-induced dehydration. Aseptically grown 14-day-old plants were exposed to drought by removing the lids from the Petri dishes for up to 60 min as described in the Materials and Methods. Total RNA was extracted from the aerial parts of drought-stressed plants at the indicated time points. Transcript levels of target genes (*RD29A*, *RD29B*, *RD26*, and *BGLU18*) were determined by RT-qPCR, normalized to those of *PPR* (A) and *UBC9* (B) as reference genes, and represented as relative values compared to the level at the start of stress treatment (0 min), which was assigned a value of 1. See Figure 3B for the results when *SAND* was used as a reference gene. PCR primer sequences are provided in Supplementary Table S1. Data are means ± SD ($n = 3$), and asterisks denote significant differences between 0-min value and 30- or 60-min value in individual genes (*$P < 0.05$; **$P < 0.01$; ***$P < 0.005$; ****$P < 0.00005$; *****$P < 0.000001$; ******$P < 0.0000005$ by unpaired Student's *t*-test).

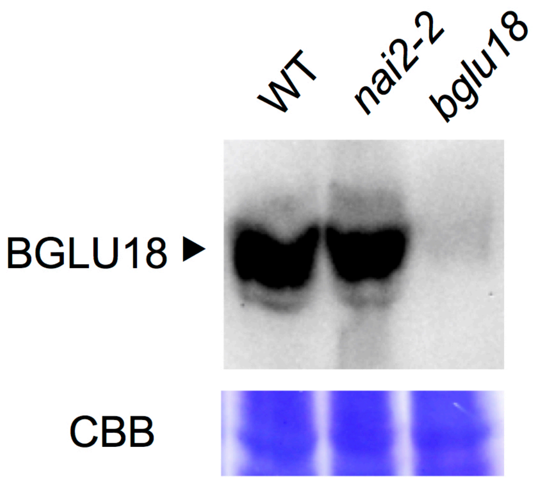

Figure S6. BGLU18 protein levels in WT, *nai2-2*, and *bglu18* plants. Immunoblotting was performed with anti-BGLU18 antibodies using proteins extracted from leaves of 14-day-old plants grown under normal conditions. CBB, Coomassie Brilliant Blue staining as a control for protein loading.

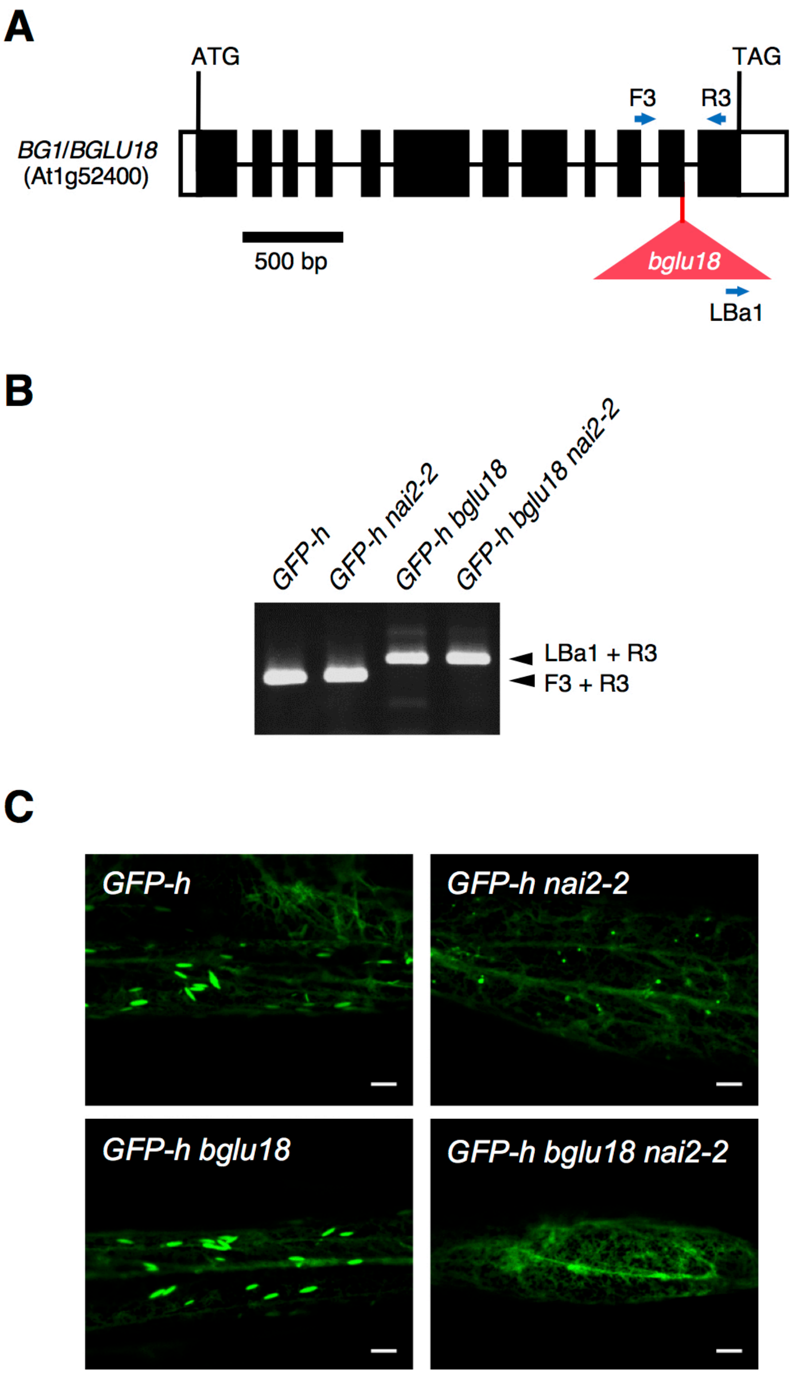

Figure S7. Genotyping and phenotyping of the *bglu18 nai2-2* double mutant. The homozygous *bglu18* (SALK_075731C; Ogasawara *et al.*, 2009) and *nai2-2* (SALK_005896; Yamada *et al.*, 2008) mutants were crossed to obtain the *bglu18 nai2-2* double mutant. All mutants expressed *GFP-h* to visualize ER/ER bodies. (A) Diagram of the T-DNA insertion in *BG1/BGLU18* (At1g52400) in the *bglu18* mutant. Arrows denote PCR primers. (B) PCR-based genotyping of the double mutant using primers specific to *BGLU18* (F3 and R3) and the left border sequence of T-DNA (LBa1). The primer sequences are listed in Supplementary Table S1. (C) Representative GFP fluorescence images of leaf petiole epidermal cells. Scale bars = 10 μm.

Table S1. Primers used in this study.

AGI[a]	Gene symbol[b]	Direction	Sequence (Designation)	Use
At1g52400	*BG1*/*BGLU18*	Forward	5′-CACCATGGTGAGGTTCGAGAAGGTT-3′ (F1)	*mRFP-BGLU18* fusion construct (for signal peptide)
		Reverse	5′-AAATTTGTCAGGCAGGCCTGCACC-3′ (R1)	*mRFP-BGLU18* fusion construct (for signal peptide)
		Forward	5′-AGCAGATTAAACTTCCCTGAAGGC-3′ (F2)	*mRFP-BGLU18* fusion construct (for mature polypeptide)
		Reverse	5′-CTAGAGTTCTTCCCTCAGCTTGG-3′ (R2)	*mRFP-BGLU18* fusion construct (for mature polypeptide)
		Forward	5′-GGCGACCCAGAAGTTATCAT-3′ (F3)	PCR genotyping
		Reverse	5′-GAATACCATTTGCCCGAAAC-3′ (R3)	PCR genotyping
		Forward	5′-TGAGTGGCAAGATGGGTACA-3′	RT-qPCR
		Reverse	5′-TCAGCTTGGAGGTTGGAAAC-3′	RT-qPCR
At4g04955	*ALN*	Forward	5′-CCTTTATGTGCCCTTCAGGA-3′ (F4)	PCR genotyping
		Reverse	5′-GGCCTATCACTCCACCAAGA-3′ (R4)	PCR genotyping
At5g52310	*RD29A*	Forward	5′-AGGAACCACCACTCAACACA-3′	RT-qPCR
		Reverse	5′-ATCTTGCTCATGCTCATTGC-3′	RT-qPCR
At5g52300	*RD29B*	Forward	5′-ACGAGCAAGACCCAGAAGTT-3′	RT-qPCR
		Reverse	5′-AGGAACAATCTCCTCCGATG-3′	RT-qPCR
At4g27410	*RD26*	Forward	5′-AGTTCGATCCTTGGGATTTG-3′	RT-qPCR
		Reverse	5′-ACCCGTTGCTTTCCAATAAC-3′	RT-qPCR
At1g62930	*PPR*	Forward	5′-GAGTTGCGGGTTTGTTGGAG-3′	RT-qPCR (as a reference)
		Reverse	5′-CAAGACAGCATTTCCAGATAGCAT-3′	RT-qPCR (as a reference)
At2g28390	*SAND*	Forward	5′-AACTCTATGCAGCATTTGATCCACT-3′	RT-qPCR (as a reference)
		Reverse	5′-TGATTGCATATCTTTATCGCCATC-3′	RT-qPCR (as a reference)
At4g27960	*UBC9*	Forward	5′-TCACAATTTCCAAGGTGCTGC-3′	RT-qPCR (as a reference)
		Reverse	5′-TCATCTGGGTTTGGATCCGT-3′	RT-qPCR (as a reference)
–	*mRFP*	Forward	5′-ATGGCCTCCTCCGAGGACGTCATCA-3′ (FW)	*mRFP-BGLU18* fusion construct
		Reverse	5′-GGCGCCGGTGGAGTGG-3′ (RE)	*mRFP-BGLU18* fusion construct
–	T-DNA	–	5′-TGGTTCACGTAGTGGGCCATCG-3′ (LBa1)	PCR genotyping

[a] *Arabidopsis thaliana* gene identifier (AGI) codes assigned according to the guidelines for nomenclature used for Arabidopsis genes (https://www.arabidopsis.org/portals/nomenclature/guidelines.jsp).
[b] Gene symbol, as provided by The Arabidopsis Information Resource (TAIR; release 10; https://www.arabidopsis.org/), except for *mRFP* and T-DNA of *Agrobacterium tumefaciens*.

Table S2. Mass spectrometry settings used for LC-ESI-MS/MS analysis of ABA in negative mode.

Analyte	Retention time on LC (min)	Molecular ion	Precursor ion (*m/z*)	Product ion (*m/z*)	Isolation width (*m/z*)	Ionization voltage (V)	Collision energy (eV)
ABA	5.5	$[M-H]^-$	263.12	153.1	2	10	30
d_6-ABA	5.5	$[M-H]^-$	269.13	159.1	2	10	30

References

Nakagawa T, Suzuki T, Murata S, Nakamura S, Hino T, Maeo K, Tabata R, Kawai T, Tanaka K, Niwa Y, Watanabe Y, Nakamura K, Kimura T, Ishiguro S (2007) Improved Gateway binary vectors: high-performance vectors for creation of fusion constructs in transgenic analysis of plants. *Bioscience, Biotechnology, and Biochemistry* **71:** 2095–2100.

Ogasawara K, Yamada K, Christeller JT, Kondo M, Hatsugai N, Hara-Nishimura I, Nishimura M (2009) Constitutive and inducible ER bodies of *Arabidopsis thaliana* accumulate distinct ß-glucosidases. *Plant and Cell Physiology* **50:** 480–488.

Winter D, Vinegar B, Nahal H, Ammar R, Wilson GV, Provart NJ (2007) An “Electronic Fluorescent Pictograph” browser for exploring and analyzing large-scale biological data sets. *PLoS ONE* **2:** e718.

Yamada K, Nagano AJ, Nishina M, Hara-Nishimura I, Nishimura M (2008) NAI2 is an endoplasmic reticulum body component that enables ER body formation in *Arabidopsis thaliana*. *The Plant Cell* **20:** 2529–2540.
